# Formation of Extrachromosomal Circular DNA from Long Terminal Repeats of Retrotransposons in *Saccharomyces cerevisiae*

**DOI:** 10.1534/g3.115.025858

**Published:** 2015-12-17

**Authors:** Henrik D. Møller, Camilla E. Larsen, Lance Parsons, Anders Johannes Hansen, Birgitte Regenberg, Tobias Mourier

**Affiliations:** * Department of Biology, University of Copenhagen, DK-2200 Copenhagen N, Denmark; † Lewis-Sigler Institute for Integrative Genomics, Princeton University, New Jersey 08544; ‡ Center for GeoGenetics, Natural History Museum of Denmark, University of Copenhagen, DK-1350 Copenhagen K, Denmark

**Keywords:** transposable elements, long terminal repeats, yeast, recombination, circular DNA

## Abstract

Extrachromosomal circular DNA (eccDNA) derived from chromosomal Ty retrotransposons in yeast can be generated in multiple ways. Ty eccDNA can arise from the circularization of extrachromosomal linear DNA during the transpositional life cycle of retrotransposons, or from circularization of genomic Ty DNA. Circularization may happen through nonhomologous end-joining (NHEJ) of long terminal repeats (LTRs) flanking Ty elements, by Ty autointegration, or by LTR–LTR recombination. By performing an in-depth investigation of sequence reads stemming from Ty eccDNAs obtained from populations of *Saccharomyces cerevisiae* S288c, we find that eccDNAs predominantly correspond to full-length Ty1 elements. Analyses of sequence junctions reveal no signs of NHEJ or autointegration events. We detect recombination junctions that are consistent with yeast Ty eccDNAs being generated through recombination events within the genome. This opens the possibility that retrotransposable elements could move around in the genome without an RNA intermediate directly through DNA circularization.

The genome of baker’s yeast, *Saccharomyces cerevisiae*, contains five families of retrotransposable elements (Ty1–Ty5) that, combined, constitute approximately 3% of the relatively compact *S. cerevisiae* genome (Kim *et al.* 1998; Carr *et al.* 2012). Elements from the Ty1–4 families are located predominantly upstream of RNA polymerase III transcribed genes, and in close proximity to tRNA genes (< 750 bp) (Chalker and Sandmeyer 1992; Devine and Boeke 1996; Bridier-Nahmias *et al.* 2015), whereas Ty5 elements display a preference for telomeric regions (Voytas and Boeke 1992; Zou and Voytas 1997). Phylogenetic analyses of Ty element sequences have revealed that putative active elements are found for all families except the fixed, and apparently inactive, Ty5 family, and the latest suggested family, Ty3p, a Ty3-like element related to the *S. paradoxus* Ty3p (Carr *et al.* 2012). Ty1 and Ty2 elements are closely related and, together with Ty4 and Ty5, they belong to the Ty1-copia group, whereas Ty3 belongs to the Ty3-gypsy group (Carr *et al.* 2012; Curcio *et al.* 2015; Sandmeyer *et al.* 2015). Both groups belong to the class of long terminal repeat (LTR) retrotransposable elements that are named for the presence of LTRs at their 5′ and 3′-ends, and are functionally and structurally related to retroviruses (Mount and Rubin 1985; Havecker *et al.* 2004). 

Retrotransposon RNA is the normal intermediate for Ty transpositions (Boeke *et al.* 1985; Havecker *et al.* 2004). The life cycle of retrotransposons initiates by transcription from the 5′-LTR sequence, and continues to the 3′-LTR end, producing a polycistronic mRNA that encodes proteins required for transposition, such as reverse transcriptase and integrase (Voytas and Boeke 1992; Kim *et al.* 1998). Full-length mRNAs are transported to the cytosol where reverse transcription takes place. This results in double-stranded DNA that, at one stage, is circularized through base pairing between the two terminal single-stranded LTR sequences (Figure 1) (Rabson and Graves 1997; Telesnitsky and Goff 1997). By DNA synthesis, the LTR sequence finally becomes linearized with identical flanking, double-stranded LTR sequences. Hence, when the linear DNA is imported into the nucleus for genomic integration (for review, see Garfinkel 2005), the two LTR sequences will be identical but will, over time, accumulate mutations such as single nucleotide polymorphisms (SNPs) (SanMiguel *et al.* 1998). 

**Figure 1:**
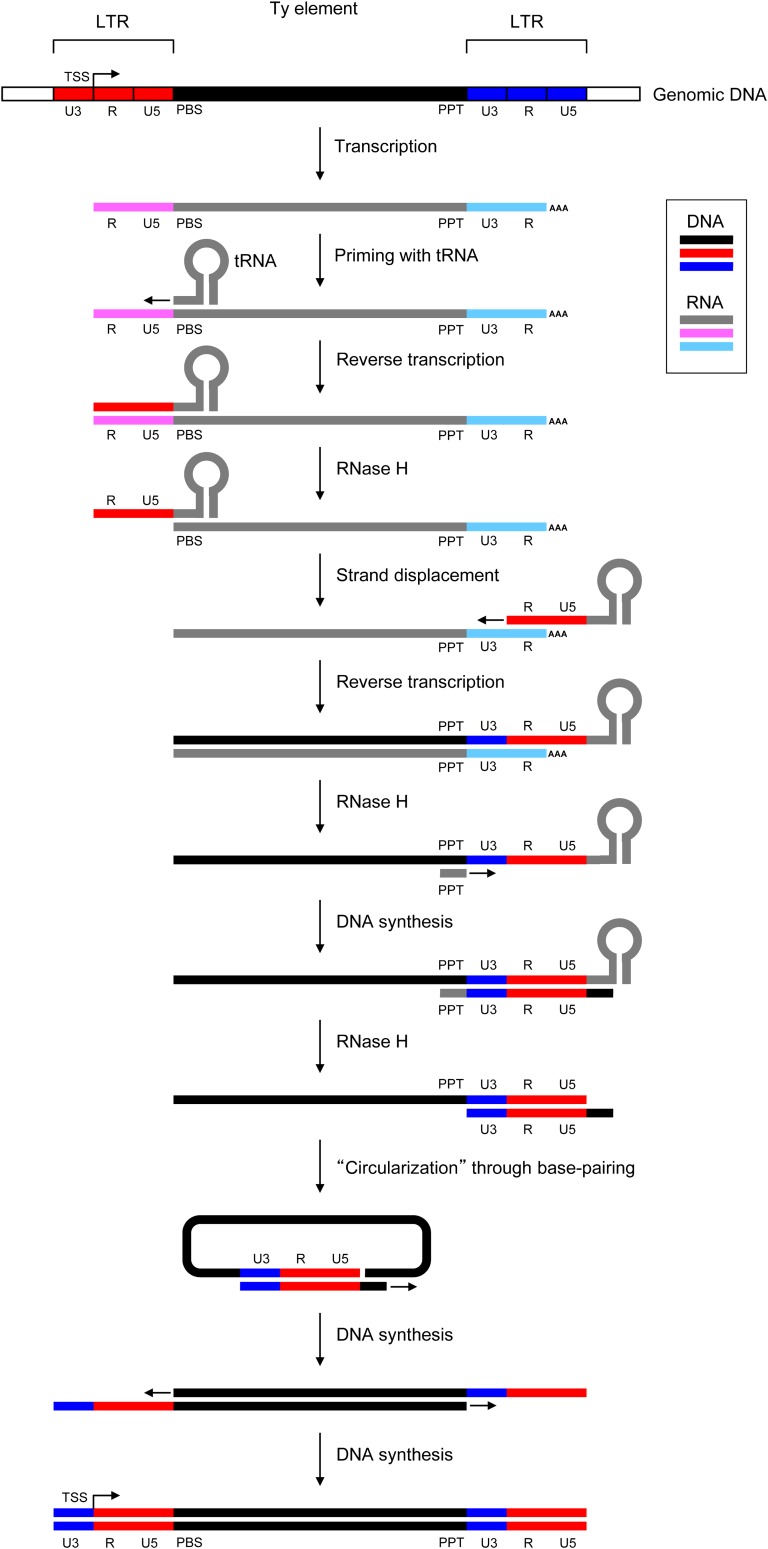
Overview of the life cycle of an LTR retrotransposable element. The transcription of a Ty element is followed by priming with tRNA, reverse transcription leading to a transcribed U5-R region (red), formation of single-stranded DNA by RNase H and strand displacement. Upon a second round of reverse transcription, a U3 region is generated from the 3′ LTR (blue). After additional steps by RNase H and subsequent DNA synthesis, a circular intermediate primes the final DNA synthesis of the Ty element through base-pairing, and the DNA circle reopens for final completion of the linear transposable Ty element by DNA synthesis. The figure is adopted and modified from Telesnitsky and Goff (1997).

The majority of genomic Ty sequences in yeast exist as solo LTR sequences (Carr *et al.* 2012). Several models such as nonallelic or unequal crossover between direct repeats can explain how solo-LTRs may form (see Liefshitz *et al.* 1995; Mieczkowski *et al.* 2006 for references). However, solo LTRs are also suggested to result from ectopic recombination between adjacent LTR sequences on the same chromatid, leaving a solo LTR excision remnant on the chromosome, and potentially a deleted full length Ty element in the form of an extrachromosomal [1-*LTR-Ty^circle^*] (Farahbaugh and Fink 1980; Jordan and McDonald 1999a, 1999b). Examples of extrachromosomal circular DNA (eccDNA) formation by recombination between solo LTRs and full-length Ty elements (Figure 2, scenarios VI and VII) have been reported (Gresham *et al.* 2010, Libuda and Winston 2006; Møller *et al.* 2015). A significant reduction in intrachromatid recombination, and the formation of [*Ty^circles^*] was reported in *rad52* and *rad1* yeast mutants (Liefshitz *et al.* 1995; Nevo-Caspi and Kupiec 1996), as well as in human cells with *MRE11*-gene mutations (Kilzer *et al.* 2003), suggesting that DNA circularization is mediated by DNA repair proteins (Liefshitz *et al.* 1995; Ivanov *et al.* 1996; Nevo-Caspi and Kupiec 1996). However, double-stranded eccDNA from retroviruses and LTR elements can be generated through several mechanisms other than intrachromatid recombination between the 5′ and the 3′ LTR. Alternatives are the nonhomologous end-joining (NHEJ) of linear Ty DNA [2-*LTR-Ty^circle^*] and linear Ty DNA integration into itself (autointegration). Ty autointegration can lead to either an inverted [2-*LTR^inverted^-Ty^circle^*] structure, or form two minor truncated circles [1-*LTR-Ty^truncated circle^*] (Figure 2, scenario III+IV) (Shoemaker *et al.* 1980; Farnet and Haseltine 1991; Hong *et al.* 1991; Lee and Craige 1994; Garfinkel *et al.* 2006). It is unknown to what extent these different mechanisms are responsible for the formation of Ty eccDNAs.

**Figure 2:**
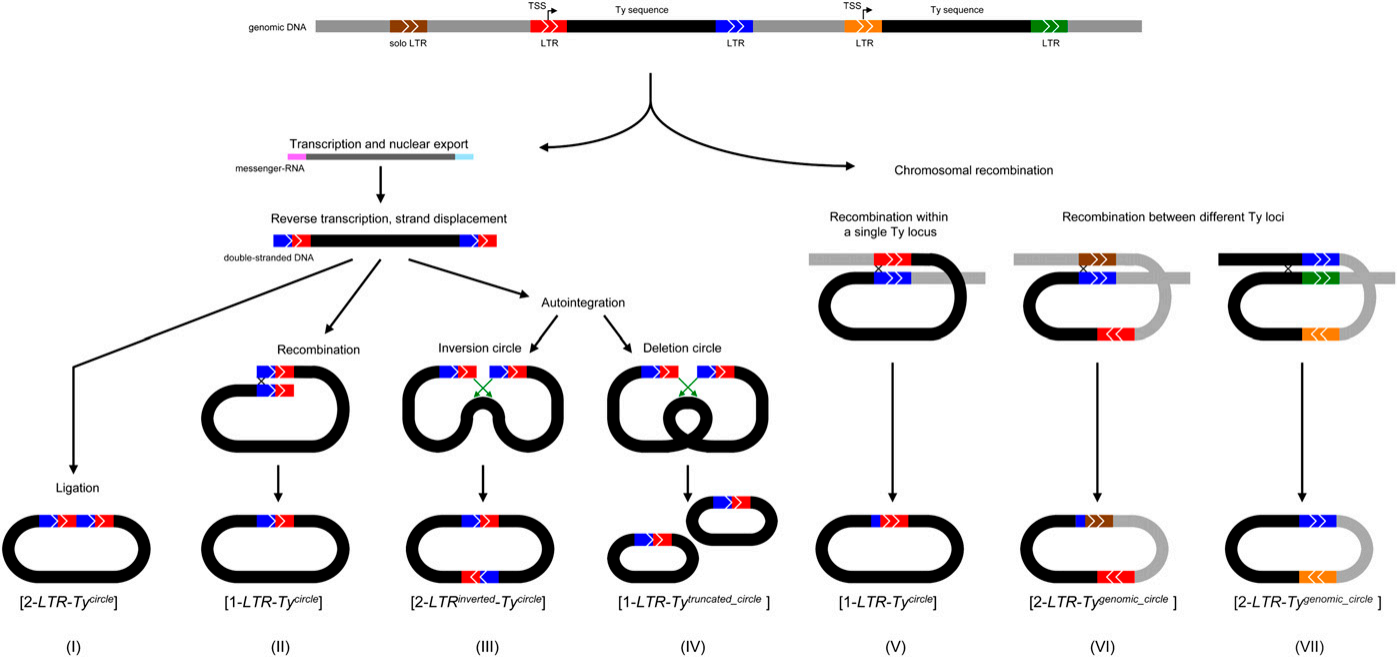
Schematic overview of the different paths to Ty eccDNA formation. For scenarios I–IV (see Figure 1), sequence reads covering the LTR regions from eccDNAs will all display an apparent breakpoint at the site corresponding to the transcription start site (TSS, top panel). Ligation (scenario I) mediated by nonhomologous end-joining (NHEJ) of 3′- and 5′-LTR ends (Kilzer *et al.* 2003). Recombination of LTRs (scenario II) mediated by homologous recombination (Farnet and Haseltine 1991). Auto-integration (scenarios III and IV) caused by double-stranded Ty DNA insertion into its own sequence rather than into the nuclear genome (indicated by green arrows) (Garfinkel *et al.* 2006). For scenarios V, VI and VII (Farahbaugh and Fink 1980; Jordan and McDonald 1999a; Libuda and Winston 2006; Møller *et al.* 2015), intrachromatid recombination and crossover events is envisioned at any position along the entire length of the LTR sequences of Ty eccDNAs.

We recently conducted a highly sensitive, whole genome analysis of eccDNA in the yeast, *S. cerevisiae* S288c, eliminating linear chromosomal DNA by extensive exonuclease treatment. We found that eccDNA is derived from numerous sites across the entire genome, and showed that LTRs in flanking regions of unique eccDNA sequences were significantly overrepresented (Møller *et al.* 2015). Here, we present an in-depth analysis of eccDNAs stemming from Ty elements. We find strong support for the presence of [*Ty^circles^*] produced through recombination between genomic LTRs, and find no bridging reads that could indicate the existence of [2-*LTR-Ty^circles^*]. 

## Materials and Methods

### Strains and eccDNA datasets

All strains used for preparing eccDNA datasets are in the S288C background **(**Lindstrom and Gottschling 2009; Møller *et al.* 2015). EccDNA datasets (R1, R3, Z1, Z3, S1, and S2 samples) were from a previous study (Møller *et al.* 2015), and have been deposited in the European Nucleotide Archive (primary accession no. PRJEB6368, secondary accession no. ERP005892). EccDNA datasets (B02–B05 samples) have been deposited in the European Nucleotide Archive (primary accession no. PRJEB11638, secondary accession no. ERP013036). 

### EccDNA preparation from biological B02–B05 samples

For each sample, cells were picked from a single yeast colony to inoculate an overnight culture in complete medium (YPD) at 30°. Next day each culture was diluted to 0.2 (OD_600_), and regrown in fresh complete medium to 0.8 (OD_600_). Cells were washed twice in PBS and diluted in PBS to 0.7 × 10^7^ cells/ml (12 ml in total). A fraction of 1 × 10^7^ cells was resuspended in PBS Sulfa-NHS-LC-biotin (Pierce Chemical, Rockford, IL) for 30 min. Cells were pelleted, fixed in 70% ethanol and subsequently counted after rehydration in PBS, using a hemocytometer. For each sample, 1 × 10^6^ cells were pelleted and frozen on liquid nitrogen (N_2_). Enrichment of eccDNAs from 1 × 10^6^ yeast cells were performed as described (Møller *et al.* 2015) with minor changes: Cell membranes were disrupted by 10 units of zymolyase (USBiological) for 1.5 hr at 35°. Zymolyase was inactivated at 60° for 5 min. At the eccDNA enrichment step by column chromatography, samples were incubated at –20° for 45 min after DNA precipitation, and then centrifuged at 9788 × *g* for 30 min at 2°, followed by 70% cold ethanol wash and recentrifugation at 9788 × *g* for 5 min at 2°. The air-dried DNA was dissolved in 19 µl of sterile water, and treated with *Swa*1 in two cycles (2 × 2 FastDigest units, Thermo Scientific), each cycle of 1 hr at 37° (total volume 25 µl), and heat-inactivation for 15 min at 65°. To remove all linear chromosomal DNA, four units of exonuclease (Plasmid-Safe ATP-dependent DNase, Epicentre) was added to the reaction at 37° (reaction volume 40 µl), and, each 24 hr, additional ATP, buffer, and 2.5 units exonuclease was added. After digestion for 6 d, enzymes were heat-inactivated for 30 min at 70°. The liquid (∼47 µl) was evaporated to half volume on a vacuum centrifuge (MAXI-DRY LYO, Heto), and 10 µl of the volume was treated with REPLI-g Mini (Qiagen) for 40 hr.

### Sequence reads analysis

The genome sequence of the S288c reference strain was downloaded from the *Saccharomyces* Genome Database (www.yeastgenome.org). Ty annotations were taken from reference Carr *et al.* (2012). A few instances of overlapping annotations were manually split. Coordinates used are provided in Supporting Information, Table S3.

Reads were mapped to the S288c reference genome and the 2μ plasmid using BWA version 0.7.5a with default parameters (Li and Durbin 2010). Duplicate reads were removed using the rmdup function in samtools (http://samtools.sourceforge.net/). For each read, up to a 1000 mapping coordinates were obtained from BWA and all reported coordinates were considered. Read coverage across genomic positions was recorded using custom scripts and the bedtools suite (Quinlan and Hall 2010). Weighted read coverage was calculated by assigning each read a coverage value of 1/(total number of mappings for read) at each genomic position covered.

### Coverage of different Ty families

The mapping positions for each read mapping to a Ty sequence were resolved with respect to the families to which it maps. For example, a read may map to 10 loci, eight being Ty1, two being Ty2. This read will be counted as the intersection between Ty1 and Ty2. The expected coverage of different Ty families was estimated by extracting all genomic sequences annotated as Ty and constructing all possible 142-nucleotide long subsequences from this set. These sequences were then mapped onto the genome and the coverage was calculated. 

Telomeric regions and genomic regions harboring ribosomal RNA genes are previously shown to produce high levels of eccDNAs (Horowitz and Haber 1985; Sinclair and Guarente 1997; Park *et al.* 1999; Cohen *et al.* 2003; Cesare *et al.* 2008). These regions were identified from the annotation at *Saccharomyces* Genome Database and were excluded from the analysis of read coverage distribution. The observed distribution when including these regions is provided in Figure S4.

### Coverage across Ty elements

The expected coverage across full-length Ty1 elements of eccDNAs that were generated between LTRs from the same Ty element was assessed by extracting the sequence from the midpoint of the 5′ LTR to the midpoint of the 3′ LTR of all Ty1 elements annotated as full-length. From this sequence, all possible 142-nt long subsequences were generated, including subsequences from the concatenation between end and start of the LTRs. These were mapped onto the reference genome, and the weighted coverage across all Ty elements was calculated. 

To obtain the expected coverage of eccDNAs that were generated by ligation of extrachromosomal Ty dsDNA, the entire sequence of all full-length Ty elements including both flanking LTRs was extracted, and the above procedure was repeated. Similarly, the expected coverage of eccDNAs that were exclusively generated between LTR, and not residing in the same Ty elements, was obtained by extracting all sequences between annotated LTR sequences (both solo LTRs and LTRs residing in full-length elements), if these were separated by a minimum of 10 bp and a maximum of 30,000 bp.

### Searching for mosaic Ty sequences in eccDNAs

All sequence reads were split in two parts, consisting of the first and last 50 nucleotides, respectively. These parts were mapped onto the genomic sequence of all 32 full-length Ty1 elements. Only instances where at least one of the parts from a read mapped to the LTR part of the Ty elements were considered. Cases where read parts mapped in a pattern not consistent with their origin from a single read sequence, *i.e.*, downstream read part mapping upstream or parts mapping in incorrect orientations, were extracted and shown in Figure S2.

### Breakpoint assessment

LTR sequences from the 32 annotated full-length Ty1 elements were aligned pairwise, *i.e.*, the upstream LTR from a given Ty element was aligned against the downstream LTR from the same element. From each of these alignments, all possible chimeric LTR sequences were generated in the following manner: the first chimeric sequence consists of the nucleotide from the downstream LTR at the first alignment position, and the nucleotides from the upstream LTR at all the rest of the alignment positions; the second chimeric sequence consists of the nucleotides from the downstream LTR at the first two alignment positions, and the nucleotides from the upstream LTR at all the rest of the alignment positions. This was repeated until the chimeric sequence consisted entirely of the downstream LTR with the exception of the last base, which was from the upstream LTR. This was done for all 32 pairs of LTRs from annotated full-length Ty1 elements, and all sequence reads were then mapped onto the entire collection of chimeric sequences. Reads that could be mapped to any chimeric sequence, and could not be mapped to the unmanipulated genome, were considered as reads supporting apparent breakpoints. To ensure that the informative positions in sequence reads supporting apparent breakpoints were not artifacts from the relatively high error rate of next-generation sequencing techniques, we carefully checked the base calling quality scores (Figure S5).

## Results and Discussion

### Ty sequences in eccDNAs

Sequence reads (average read length > 141 nucleotides, Figure S1) were retrieved from a purified and enriched eccDNA collection, obtained from two S288c clonal yeast populations of 1 × 10^10^ cells (Møller *et al.* 2015). The 75–80 million reads from the two samples (samples S1 and S2) were remapped to the S288c reference genome, and the 2μ plasmid sequence. We found that reads mapping to annotated Ty sequences constituted 6.1% and 10.7% of the total reads mapping to the nuclear genome in samples S1 and S2, respectively (Table 1). 

**Table 1:** Read sequences in samples

		Sample S1	Sample S2
Total reads		73,049,571	81,221,653
Mapped reads	Nuclear genome	1,742,894	1,851,060
	Mitochondria	190,572	293,715
	2μ-plasmid	64,502,069	66,793,292
Nonclonal reads	Nuclear genome	122,006	124,464
	Ty sequences	7481	13,318
	(Percent Ty)	(6.1%)	(10.7%)

**Figure 3:**
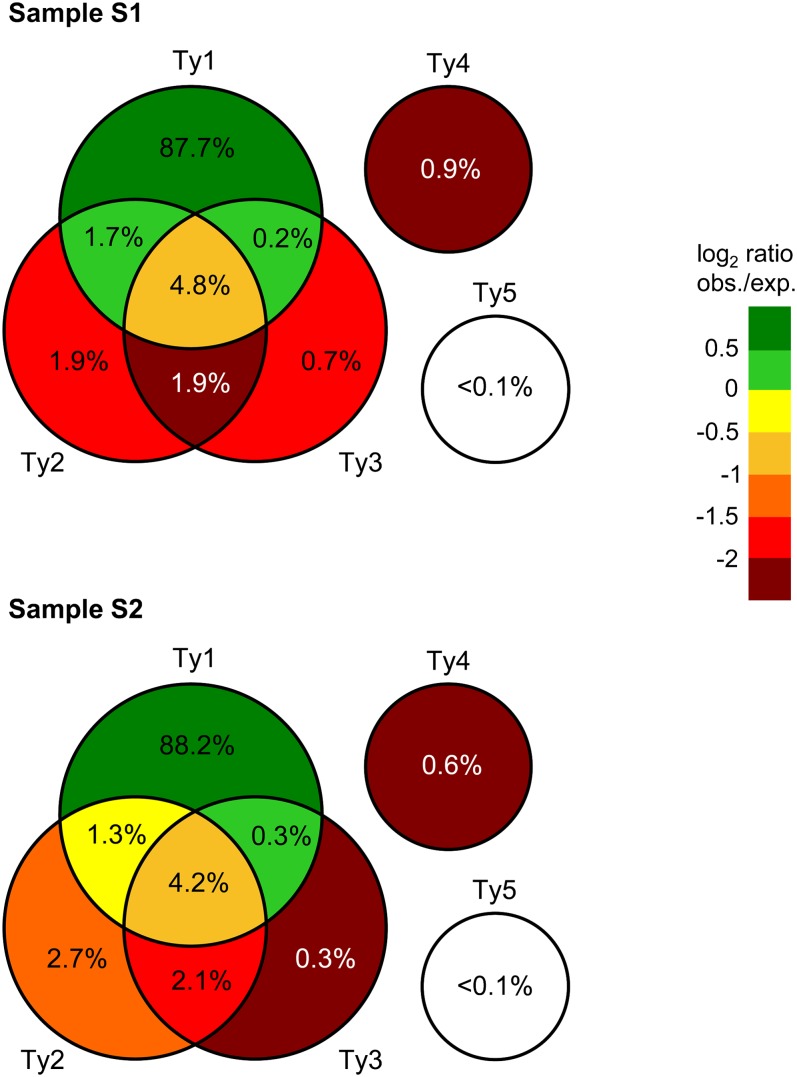
Venn diagrams of per cent reads mapping to different Ty families. Values are shown as percentages of the total number of reads mapping to annotated Ty1–Ty5 sequences for sample S1 and S2. Values below 0.1% are omitted from the diagram, but indicated for Ty5. The diagram is colored according to the log2 ratio between the observed and expected percentage of reads mapping to a given intersect (see *Materials and Methods*).

**Figure 4:**
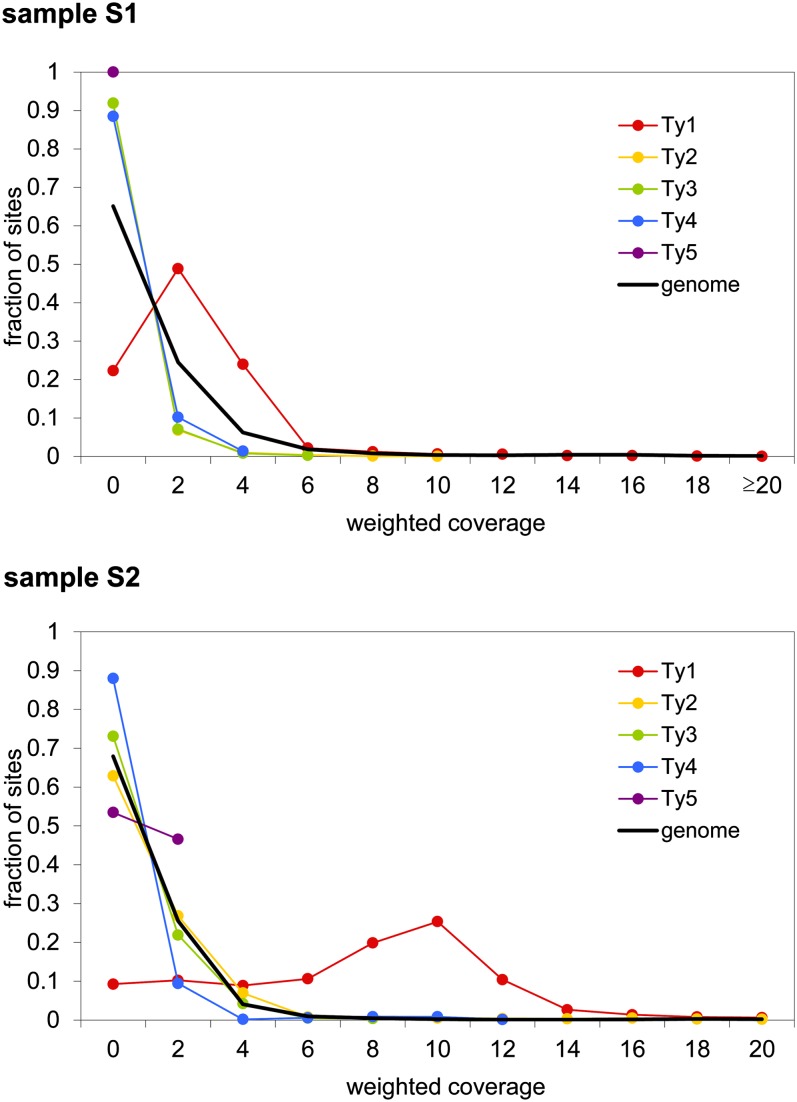
Distribution of read coverage at Ty elements *vs.* other genomic sites. For all genomic sites for a given feature, *e.g.*, a family of Ty elements, the number of mapped reads was recorded for each of the two samples, S1 and S2. Weighted coverage values for all genomic sites were sorted according to bins on the *x*-axis, and the fraction of sites falling in the bins is shown on the *y*-axis. The weighted coverage at all other genomic sites is shown as black lines (genome).

Conventionally, read sequences that map to multiple genomic locations are discarded prior to read coverage calculations. However, the redundancy of transposable elements necessitates alternative calculations (Day *et al.* 2010; Mourier 2011; Criscione *et al.* 2014). Here, the mapping of reads was weighted, meaning that multiple genomic sites mapped by the same read were counted as 1/(total number of read-mapping sites). Nearly 90% of the reads that mapped to Ty sequences mapped exclusively within sequences from the Ty1 family, which is approximately 1.5 times higher than expected from a random distribution (Figure 3). We plotted the distribution of read coverage across all genomic positions annotated as Ty sequences, and compared it to the distribution of the remaining genomic positions. We found a distinct high coverage for Ty1 compared to the other Ty2-5 elements, suggesting that at least Ty1 sequences are abundantly present on eccDNAs (Figure 4). The prevalence of Ty1 eccDNAs is likely caused by the high abundance of Ty1 full elements in the *S. cerevisiae* genome (32 out of 51 full-lengths elements belong to Ty1, Table S3), hence a much higher likelihood for picking this particular group in our study. Moreover, we expect that phi29 amplification of eccDNA results in a bias toward more common eccDNA, amplifying rare eccDNAs to a lesser degree (Møller *et al.* 2015). The redundancy of Ty sequences makes it hard to address exactly from which genomic loci the eccDNAs are derived from. To get a rough estimate of the number of full-length Ty genomic elements contributing to the pool of eccDNAs, we recorded if full-length Ty loci were covered by reads along at least 99.5% of their entire sequence, and if any reads were mapping uniquely to their genomic sequences. No full-length Ty3, Ty4, or Ty5 elements, and only a single full-length Ty2 element fulfilled the criteria (albeit in both samples, Table S1). Of the Ty1 elements in samples S1 and S2, 11 and 18, respectively, fulfilled the criteria (Table S1), and would—based on this—therefore be expected to produce eccDNAs. An additional four Ty1 elements in sample S1 and three elements in sample S2 were covered along their entire sequence but did not contain any unique 142-mer sequences (a typical length of sequence reads, Figure S1), and were therefore unlikely to harbor any uniquely mapping reads. These elements could thus potentially generate eccDNAs but we are unable to assess this. Again, these figures are rough estimates but suggest that eccDNAs are generated from multiple Ty loci, and predominantly from Ty1 elements. The latter observation prompted us to focus exclusively on Ty1 elements in the remainder of the study.

### Boundaries of Ty eccDNAs

**Figure 5:**
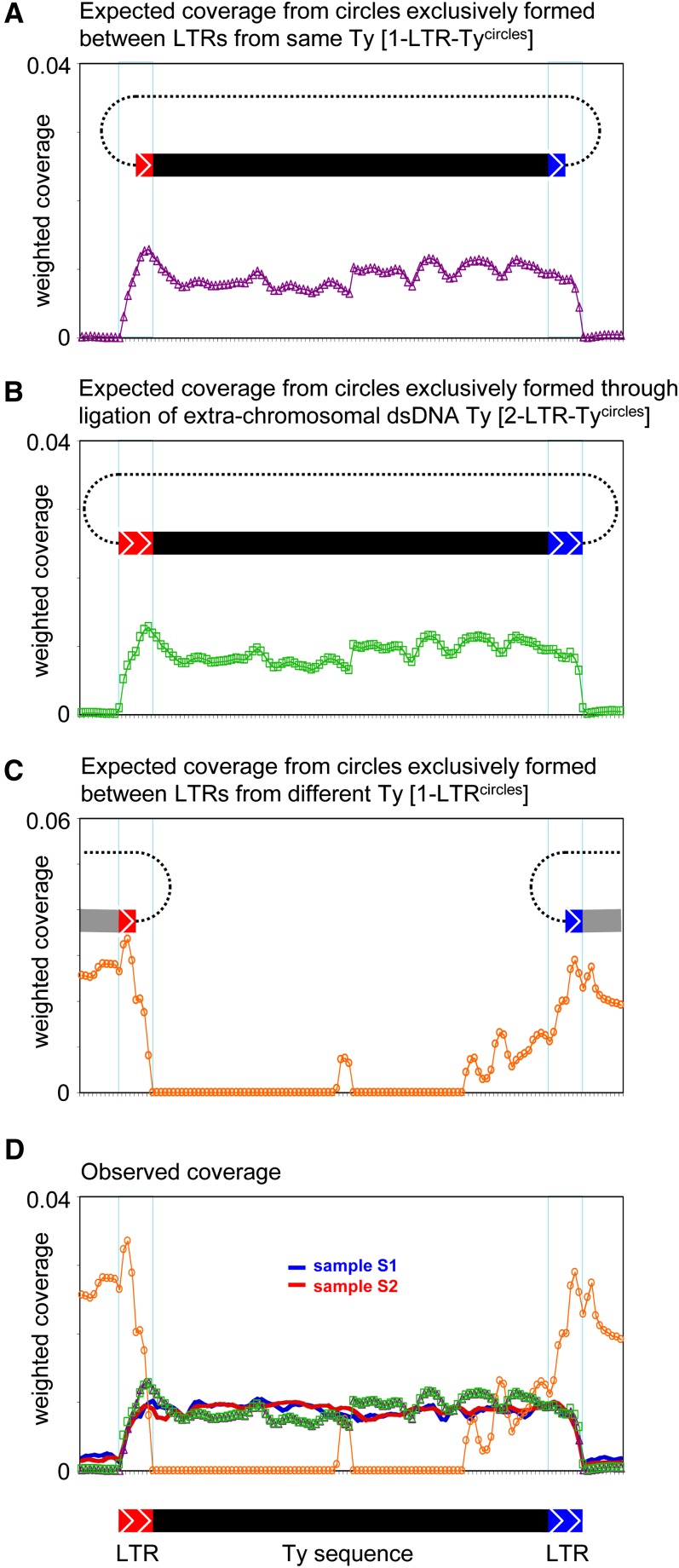
Expected and observed read coverage across full-length Ty elements. For each annotated full-length Ty element, weighted coverage was recorded in ten 50-bp windows up- and downstream of the element, and in 50-bp windows inside the entire Ty element. The represented plots represent the average of 32 annotated full-length Ty elements. (A) *In silico* genomic mapping of expected coverage of full-length Ty elements based on reads extracted from all putative [1-LTR-*Ty^circles^*]. (B) *In silico* genomic mapping as in (A), but using reads extracted from all putative [2-LTR-*Ty^circles^*]. (C) Expected weighted read coverage of [1-LTR-*Ty^circles^*] formed between LTRs of different Ty elements, based on *in silico* genomic mapping of reads extracted from putative eccDNAs with an LTR. (D) Combined plot of A–C together with the observed weighted read coverage of Ty1 elements in samples S1 and S2, respectively. In plots A–C, a schematic representation of the simulated circles is shown. The extent of the Ty internal and LTR sequences on the *x*-axes is shown below plot D, and the borders of the LTR sequences are indicated on each plot by thin blue vertical lines.

The presence of Ty sequences in eccDNAs may stem from either full-length Ty elements or partially included Ty sequences (scenarios I–V *vs.* VI–VII, Figure 2). We looked specifically at each of the annotated 32 full-length Ty1 elements, in an attempt to distinguish between the different proposed scenarios (Figure 2). We first recorded the average read coverage in 50-bp windows across full-length Ty1 elements, including 500 bp of upstream and downstream sequences. The expected read coverage from [1-*LTR-Ty1^circle^*] and [2-*LTR-Ty1^circle^*], formed from the same Ty element, as well as [1-*LTR^circle^*] formed from different Ty elements was simulated *in silico* and graphed (Figure 5, A–C). Ty1 weighted read coverage from sample S1 and S2 display an elevated plateau of mapped reads across full-length Ty1 elements (Figure 5D). The read coverage declines at the flanking LTR regions, supporting the notion that Ty eccDNAs consists predominantly of entire Ty elements that recombined at the LTRs (Figure 2, scenarios I–V). Though, given the redundancy of Ty1 LTRs in the genome with the majority of sequences existing as solo-LTRs, the simulated coverage does not reveal any clear difference between the two proposed types of Ty eccDNAs: [1-*LTR-Ty1^circle^*] and [2-*LTR-Ty1^circle^*] (Figure 2). 

### Identification of mosaic Ty sequences

The coverage in Figure 5 testifies to the presence of Ty sequences in eccDNA. In order to distinguish between the different proposed scenarios I, III, and IV (Figure 2), we searched for sequence reads that spanned junctions of Ty eccDNAs, and contained information about mosaic Ty elements. We searched for reads supporting the presence of mosaic Ty sequences by extracting the first and last 50 nucleotides from all sequence reads, and recorded all instances where the two subsets of reads mapped in patterns not consistent with the genomic full-length Ty1 sequences (see *Materials and Methods*). Based on the two analyzed eccDNA datasets (samples S1 and S2) we identified six apparent mosaics of Ty sequences (Figure S2). A single mosaic read was consistent with an autointegration event (scenario IV, Figure 2). None of the remaining five mosaic reads were compatible with the proposed scenarios I and III, *i.e.*, [2-*LTR-Ty^circle^*] and [2-*LTR^inverted^-Ty^circle^*] (Figure S2).

### Breakpoint analysis

To distinguish between Ty eccDNAs generated through the transpositional life cycle (scenario II, Figure 2), and by intrachromatid LTR–LTR recombination (scenario V, Figure 2), we attempted to map the genomic breakpoint regions of Ty eccDNAs. If LTR sequences (U3-R-U5) are completely identical, then no breakpoints can be assigned. However, if the two flanking LTRs contain SNPs or small indels, then the site of recombination can be deduced from the read sequences (Gresham *et al.* 2010). The circularization of extrachromosomal linear Ty DNA (scenario II) will—despite not being the result of recombination—display an apparent breakpoint at the transcription start site (TSS) situated at the fusion between the LTR U3 (blue) and the LTR R-U5 (red) sequence (Figure 1) (Rabson and Graves 1997). Consequently, breakpoints outside the U3-R border will indicate genomic intrachromatid recombination (scenario V), rather than circularization of the transposing extrachromosomal linear Ty DNA (scenario II). 

**Figure 6:**
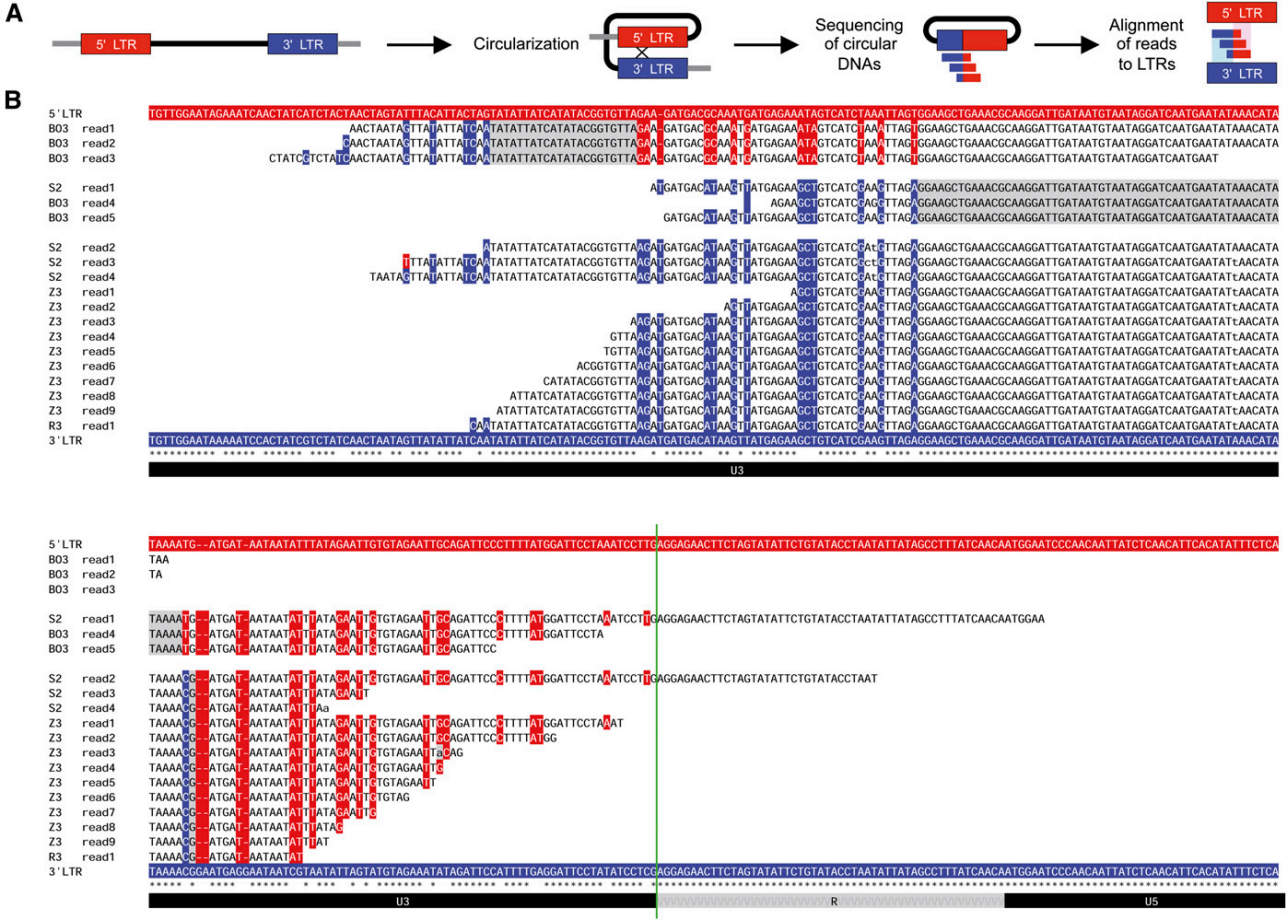
Recombination junctions within LTR sequences. (A) Schematic overview of the procedure to detect apparent breakpoints between LTR sequences. (B) Alignment of the LTR sequences from the full-length Ty1 element residing at chrX:478044–483965 shown at the top (5′ LTR, highlighted in red), and at the bottom (3′ LTR, in blue). In between are shown representative sequences from the mapped sequence reads from various samples (samples denoted to the left). Three apparent breakpoint sites are highlighted in gray. Positions in read sequences in which residues are identical to only the 5′ LTR or the 3′ LTR, but not both, are colored accordingly. Asterisks below alignment indicate positions that are identical between the two LTRs. Instances where read sequences do not match either LTRs are indicated by lower-case letters. The U3, R, and U5 regions are shown as bars underneath the alignment. The green vertical line denotes the expected apparent breakpoint site if eccDNAs were formed through circularization of linear extrachromosomal DNA. Approximate U3-R-U5 borders are deduced from Fulton *et al.* (1988) and Hou *et al.* (1994).

As any breakpoint between two LTR sequences will result in a single chimeric sequence, we constructed all possible chimeras from the 32 LTR pairs from full-length Ty1 elements, corresponding to all possible breakpoints. We then mapped all sequence reads onto these chimeric sequences (Figure 6A), and collected reads that mapped to these constructed recombination sequences but not to the reference genome. Finally, we cross-searched collected read hits for similarity to the genome using BLAST to ensure that these did not arise from genomic sequence of solo Ty1 LTRs. From the S2 dataset we found four sequence reads that map to a chimeric sequence generated from the Ty1 LTR sequences at position chrX:478044–483965. This Ty element displays a high level of sequence divergence between the two LTRs (Figure 6B). The four reads support two recombination sites upstream of the putative U3-R fusion site (Figure 6B), suggesting that this specific type of Ty1 eccDNA is not formed from circularization of linear extrachromosomal DNA (Figure 2, scenario I-IV) but rather by intrachromatid recombination between genomic Ty sequences (Figure 2, scenario V). Obviously, we cannot rule out that transcription of the Ty element is initiated upstream of the canonical U3-R transcription start site, which will result in a corresponding translocation of the apparent breakpoint. 

To gain further support, we repeated the breakpoint analysis on additional eccDNA datasets. These datasets were from a pool of *S. cerevisiae* single-gene deletion mutants, grown with and without the presence of the DNA-damaging agent Zeocin, a DNA-damaging agent known to cause double-stranded breaks (samples Z1 and Z3, and samples R1 and R3, respectively, Table S2). Zeocin was found to promote Ty eccDNA formation relative to untreated samples (Møller *et al.* 2015). Additionally, we included four *S. cerevisiae* biological replicate samples (B02–B05, Table S2). Intriguingly, we found read sequences in three out of these eight datasets, supporting the same recombination sites as in the S2 dataset as well as an additional site (Figure 6B). Based on the presence of divergent residues between the two LTRs, our breakpoint analysis indicates stretches of varying length rather than specific positions in the genome. Thus, the three annotated breakpoints found in multiple distant related samples could represent more than three specific breakpoint positions. The heterogeneity between recombining strands has been shown to halt the migration of Holliday junctions (Panyutin and Hsieh 1993), and the site of recombination has been suggested to be limited by base heterogeneity and composition (Sun *et al.* 1998; Forth *et al.* 2011). The apparent breakpoints in Figure 6, appearing after long stretches of identical sequence, are consistent with additional LTR recombination events occurring at this locus. Taken together, the chimeric sequence reads indicate at least three breakpoints at chrX:478044–483965 that are likely true hallmarks of genomic recombination between LTRs flanking a Ty1 element rather than the result of transposition.

The fact that this analysis of breakpoints reports events from only a single Ty1 locus is not surprising: if flanking LTR sequences are too identical, or have closely related copies residing elsewhere in the genome, these are not informative for our analysis, presumably limiting our analysis to a few specific Ty loci (Table S1). Notably, we also found two read sequences supporting an apparent breakpoint in another Ty loci (chrXV:117703–123628) that does not display a clear-cut switch between the upstream and the downstream LTR sequences (Figure S3 and Figure S6). 

### Conclusions

By performing a transposon-centered analysis, we found ample Ty sequences in eccDNAs, with sequences from the Ty1 family being overrepresented. Read coverage suggests that most [*Ty^circles^*] consist of entire Ty elements. Breakpoint analysis is consistent with [*Ty^circles^*] being generated by intrachromatid LTR–LTR recombination, while it should be stressed that the redundancy of LTR sequences means that we cannot assess the breakpoints for the vast majority of Ty elements. 

Maintaining highly homologous Ty sequences is not only caused by active retrotransposons but also mediated by recombination between distantly located Ty sequences, and potentially [*Ty^circles^*], causing gene conversion or chromosomal aberrations (Roeder and Fink 1982; Argueso *et al.* 2008; Jasin and Rothstein 2013). The circularization of Ty elements is likely facilitated by DNA repair processes involving sequence homology, such as homologous recombination (Gresham *et al.* 2010), or single-strand annealing (Ivanov *et al.* 1996). A proposed model for double-stranded-break repair through homologous recombination suggests that donor sequences are preferentially recruited from intrachromosomal sequences in close proximity to the break (Agmon *et al.* 2009**)**. Recombination between LTR sequences flanking a Ty element is in agreement with this. Initiation of single-strand annealing by resection of linear DNA (Lin *et al.* 1984, 1985) could potentially also produce the observed apparent breakpoints in [*Ty^circles^*]. Yet, analyses of Ty eccDNA from DNA repair defective mutants would shed further light on the formation mechanisms. In the current data, we find no signs of NHEJ events that could produce [2-LTR*-Ty^circles^*]. Given that [2-LTR*-Ty^circles^*] have previously been reported (Kilzer *et al.* 2003; Garfinkel *et al.* 2006), we speculate that they may be captured under other experimental conditions. For instance, by harvesting cells before entry to stationary phase, or by cultivating cells at a more optimal temperature for transposition, *i.e.*, 15–20° instead of 30° (Paquin and Williamson 1984; Garfinkel *et al.* 2005**)**. A lower temperature would presumably also increase the chance of recording autointegration events (scenario III and IV, Figure 2) that may be enhanced further by cultivating cells in a high salt concentration, reported to disrupt the viral nucleoprotein complex barriers of autointegration (Lee and Craigie 1994). 

Besides inflating genome size, insertion of Ty elements can have direct deleterious effects (Garfinkel *et al.* 2005), and transposition is suppressed in several ways (Jiang 2002; Garfinkel *et al.* 2003; Curcio *et al.* 2015). Yet, environmental factors may induce transposition of Ty elements (Garfinkel *et al.* 2005; Servant *et al.* 2012), as well as altering the genomic targeting of insertions (Wilke *et al.* 1989; Dai *et al.* 2007). This suggest that transposable elements may act as inducers of genetic variability under stressful conditions (Ebina and Levin 2007; Levin and Moran 2011; Mourier *et al.* 2014). Transposable elements are divided into elements that move through a copy-and-paste mechanism (class I elements, retrotransposons to which yeast LTR transposons belong), and elements that move through a cut-and-paste mechanism (class II, DNA transposons) (Kazazian 2004; Wicker *et al.* 2007). Recombination between genomic sequences and plasmids/episomes has been observed previously, resulting in circular DNAs being inserted back into the genome (Orr-Weaver *et al.* 1981; Cox *et al.* 1990; Vogt *et al.* 2014). We speculate that recombination between a genomic solo LTR sequence and Ty eccDNA could allow the movement from one genomic locus to another. If so, the class I LTR elements would be capable of moving around the genome in a manner similar to that of class II elements. This would require a target site in the form of a genomic LTR sequence, and movement would be restricted to genomic loci already harboring an LTR sequence. Nevertheless, as solo LTRs and full Ty elements display markedly different properties in terms of transcriptional activity, chromatin modulation, and enhancer activity (Ben-Aroya *et al.* 2004; Mourier and Willerslev 2010; Feng *et al.* 2013), the ability to change solo LTRs to full-length Ty elements—and vice versa—could provide a rapid and efficient means of genetic adaptation in yeast genomes.

## Supplementary Material

Supporting Information
