# Supplementary material for: Formation of Extrachromosomal Circular DNA from Long Terminal Repeats of Retrotransposons in Saccharomyces cerevisiae
Source: G3 (Bethesda). 2015 Dec 17;6(2):453–62. doi: 10.1534/g3.115.025858 (PMC4751563; doi:10.1534/g3.115.025858)
Supplement: Supporting Information [file supp_6_2_453__index.html]

Formation of Extrachromosomal Circular DNA from Long Terminal Repeats of Retrotransposons in Saccharomyces cerevisiae — Supporting Information 

# Formation of Extrachromosomal Circular DNA from Long Terminal Repeats of Retrotransposons in *Saccharomyces cerevisiae*

## Supporting Information for Møller *et al.*, 2016

**Files in this Data Supplement:**

- Figure S1 - Sequence read lengths (.pdf, 88 KB)
- Figure S2 - Graphical display of mosaic mapping patterns (.pdf, 82 KB)
- Figure S3 - Break-point example (.pdf, 102 KB)
- Figure S4 - Distribution of read coverage at Ty elements versus other genomic sites. (.pdf, 92 KB)
- Figure S5 - Base calling quality scores for reads indicative of apparent breakpoints. (.pdf, 152 KB)
- Figure S6 - Schematic overview of double Holliday junction formation during illegitimate recombination between two LTR sequences. (.pdf, 82 KB)
- Table S1 - Coverage of individual full-length Ty element loci (.pdf, 132 KB)
- Table S2 - Overview of samples (.pdf, 94 KB)
- Table S3 - Genomic coordinates of Ty sequences. (.pdf, 213 KB)
