# Supplementary material for: Formation of Extrachromosomal Circular DNA from Long Terminal Repeats of Retrotransposons in Saccharomyces cerevisiae
Source: G3 (Bethesda). 2015 Dec 17;6(2):453–62. doi: 10.1534/g3.115.025858 (PMC4751563; doi:10.1534/g3.115.025858)
Supplement: Supporting Information [file supp_g3.115.025858_FigureS1.pdf]

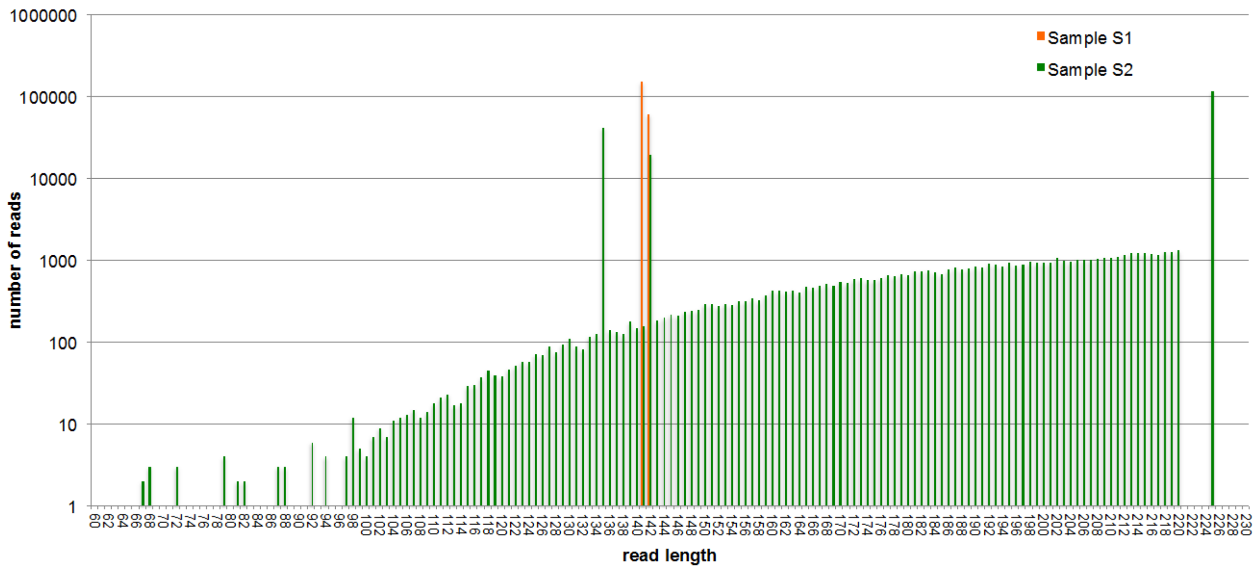

Figure S1 Sequence read lengths
The distribution of reads length shown for samples S1 and S2. The average reads length is 141.3 nucleotides for sample S1, and 193.0 nucleotides for sample S2.
