# Supplementary material for: Formation of Extrachromosomal Circular DNA from Long Terminal Repeats of Retrotransposons in Saccharomyces cerevisiae
Source: G3 (Bethesda). 2015 Dec 17;6(2):453–62. doi: 10.1534/g3.115.025858 (PMC4751563; doi:10.1534/g3.115.025858)
Supplement: Supporting Information [file supp_g3.115.025858_FigureS2.pdf]

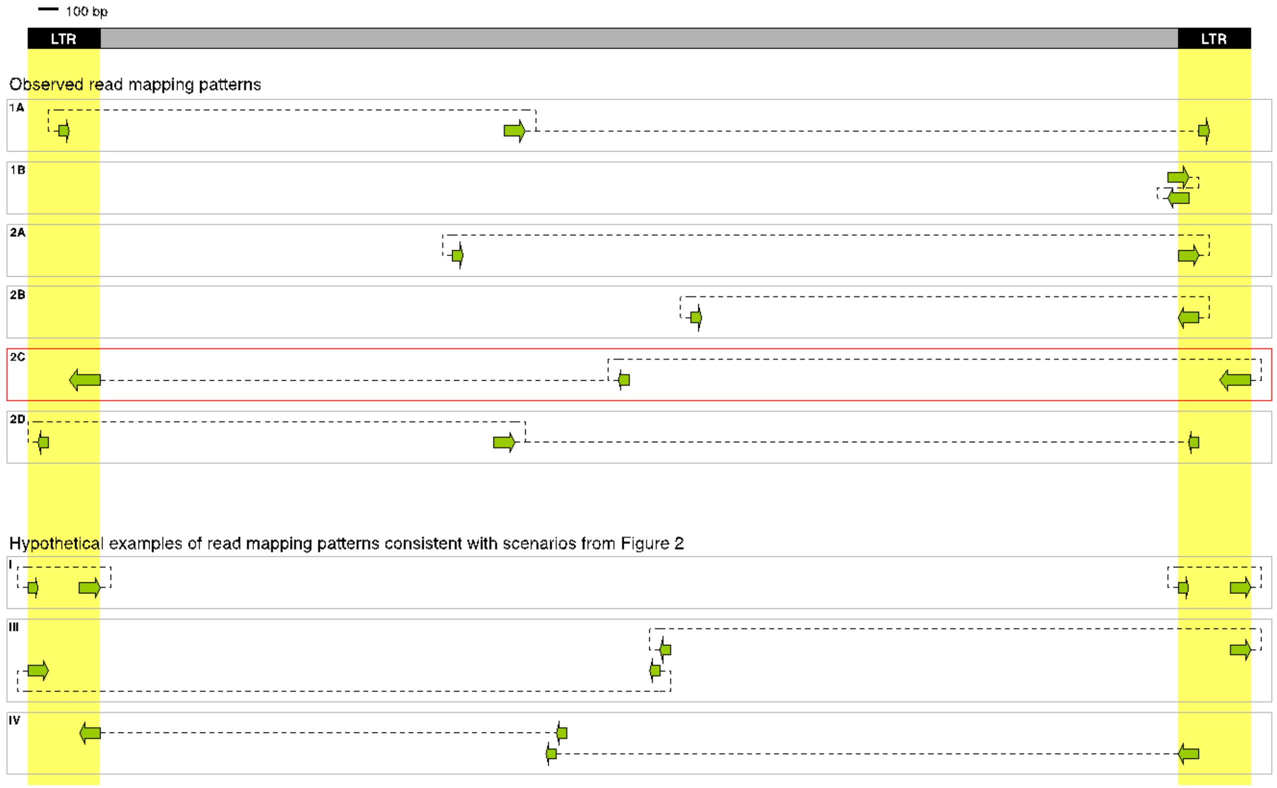

Figure S2 Graphical display of mosaic mapping patterns
Schematic depiction of split reads mapping to Ty elements. Green arrows denote the location of where the parts (first and last 50bp, respectively) map. Parts are connected by dotted lines. If one part maps to both LTR sequences (highlighted in yellow) both mapping positions are given their own arrow that is connected to the other part of the read. Parts mapping in opposite orientations result in arrows pointing in opposite directions. Top: Cases where parts from split reads map in a pattern not consistent with their origin from a single read sequence. Two cases were found in sample S1 (1A and 1B), and four cases in sample S2 (2A-D). Orientation of read parts mapping indicated by arrows. Bottom: Hypothetical examples of mapping patterns expected from different scenarios (consistent scenario indicated by roman numerical). Only observed case 2C (framed in red) is consistent with a predicted scenario.
