# Supplementary material for: Formation of Extrachromosomal Circular DNA from Long Terminal Repeats of Retrotransposons in Saccharomyces cerevisiae
Source: G3 (Bethesda). 2015 Dec 17;6(2):453–62. doi: 10.1534/g3.115.025858 (PMC4751563; doi:10.1534/g3.115.025858)
Supplement: Supporting Information [file supp_g3.115.025858_FigureS3.pdf]

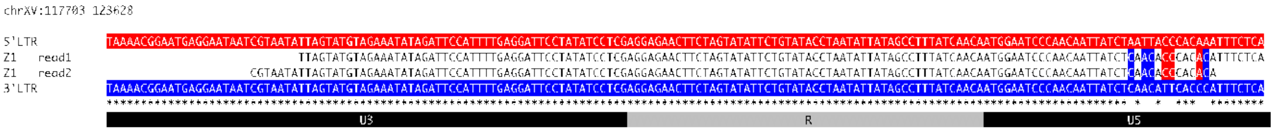

Figure S3 Break-point example

Two sequence reads not displaying a clear-cut switch between the upstream and the downstream LTR sequences are shown in a format similar to Figure 6 in main text. The LTRs are residing in a Ty element located at chrXV:117703-123628.

Considering the recombination at the molecular level, a junction is formed around the initial breakpoint site, which result in heteroduplex stretches where the two DNA-strands are derived from different non-allelic LTRs (Supplementary Fig. S6). One might speculate that this was the result of the observed pattern. The resolution of a heteroduplex sequence by the eukaryotic mismatch repair system involves the excision of long (up to several hundred base pairs) sequence fragments around the mismatch with the resulting gap subsequently being filled by polymerase and ligase activity (Fang and Modrich 1993; Giannattasio *et al.* 2010; Pena-Diaz and Jiricny 2012). Although the exact mechanism is currently unknown, such excisions would not be in agreement with the observed pattern of segments of a few bases apparently alternating between the two parental strands in the heteroduplex.

Fang, W. H., and P. Modrich, 1993 Human strand-specific mismatch repair occurs by a bidirectional mechanism similar to that of the bacterial reaction. J Biol Chem 268: 11838-11844.

Giannattasio, M., C. Follonier, H. Tourriere, F. Puddu, F. Lazzaro et al., 2010 Exo1 competes with repair synthesis, converts NER intermediates to long ssDNA gaps, and promotes checkpoint activation. Mol Cell 40: 50-62.

Pena-Diaz, J., and J. Jiricny, 2012 Mammalian mismatch repair: error-free or error-prone? Trends Biochem Sci 37: 206-214.
