# Supplementary material for: Formation of Extrachromosomal Circular DNA from Long Terminal Repeats of Retrotransposons in Saccharomyces cerevisiae
Source: G3 (Bethesda). 2015 Dec 17;6(2):453–62. doi: 10.1534/g3.115.025858 (PMC4751563; doi:10.1534/g3.115.025858)
Supplement: Supporting Information [file supp_g3.115.025858_FigureS4.pdf]

sample S1

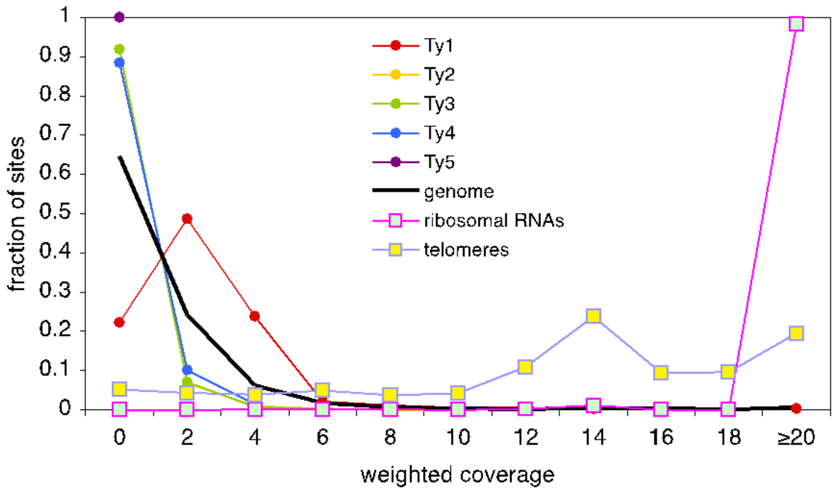

sample S2

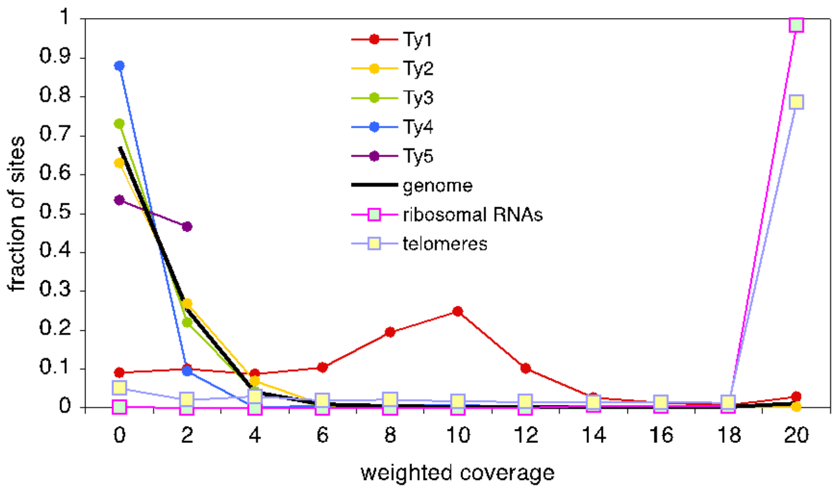

Figure S4 Distribution of read coverage at Ty elements versus other genomic sites.
Similar to figure 4 in main text but including the coverage of telomeric regions and regions harboring ribosomal RNA genes.
