# Supplementary material for: Formation of Extrachromosomal Circular DNA from Long Terminal Repeats of Retrotransposons in Saccharomyces cerevisiae
Source: G3 (Bethesda). 2015 Dec 17;6(2):453–62. doi: 10.1534/g3.115.025858 (PMC4751563; doi:10.1534/g3.115.025858)
Supplement: Supporting Information [file supp_g3.115.025858_FigureS5.pdf]

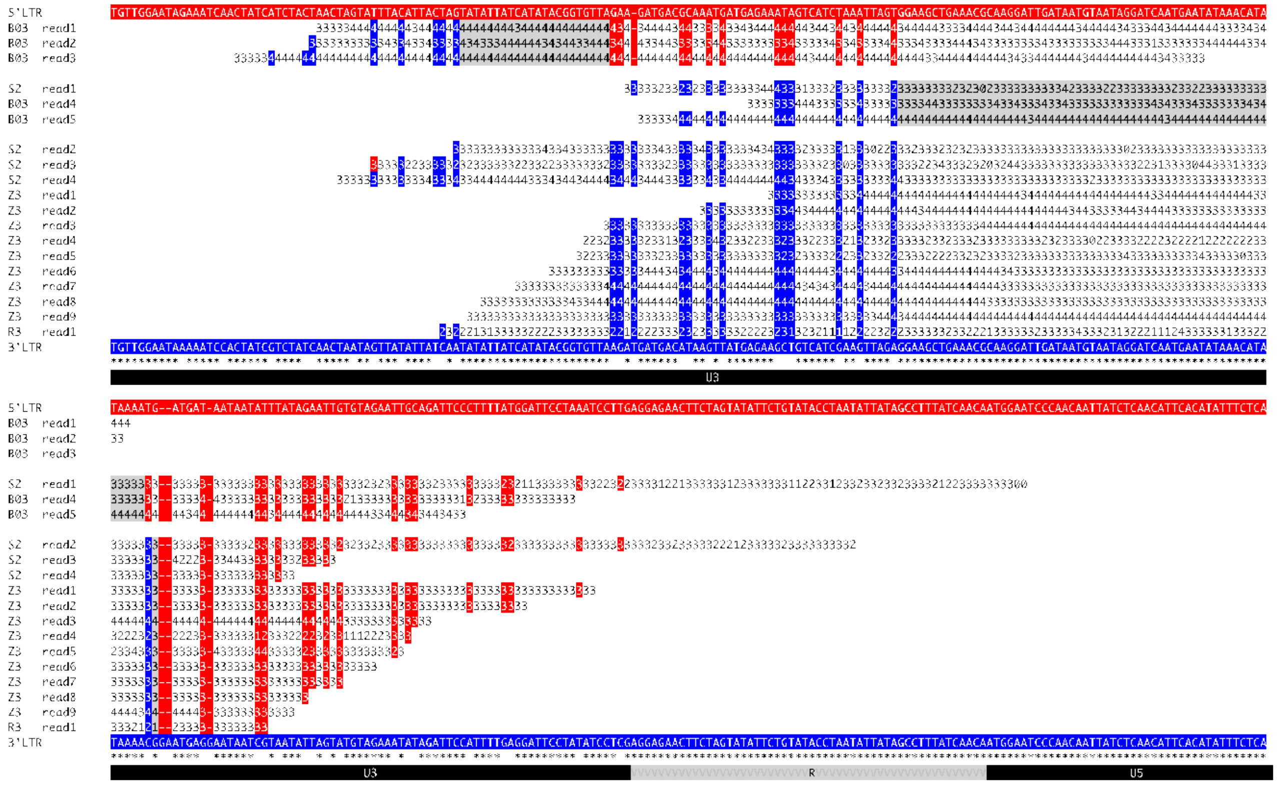

Figure S5 Base calling quality scores for reads indicative of apparent breakpoints.
Similar to figure 6 but showing quality scores rather than sequences. '4' denotes a phred score of at least 40, equivalent to a minimum accuracy of 99.99%. '3' denotes phred≥30, and accuracy 99.9%. '2' denotes phred≥20, and accuracy 99%. '1' denotes phred≥10, and accuracy 90%, '0' denotes phred <10, and accuracy less than 90%.
