# Supplementary material for: Formation of Extrachromosomal Circular DNA from Long Terminal Repeats of Retrotransposons in Saccharomyces cerevisiae
Source: G3 (Bethesda). 2015 Dec 17;6(2):453–62. doi: 10.1534/g3.115.025858 (PMC4751563; doi:10.1534/g3.115.025858)
Supplement: Supporting Information [file supp_g3.115.025858_FigureS6.pdf]

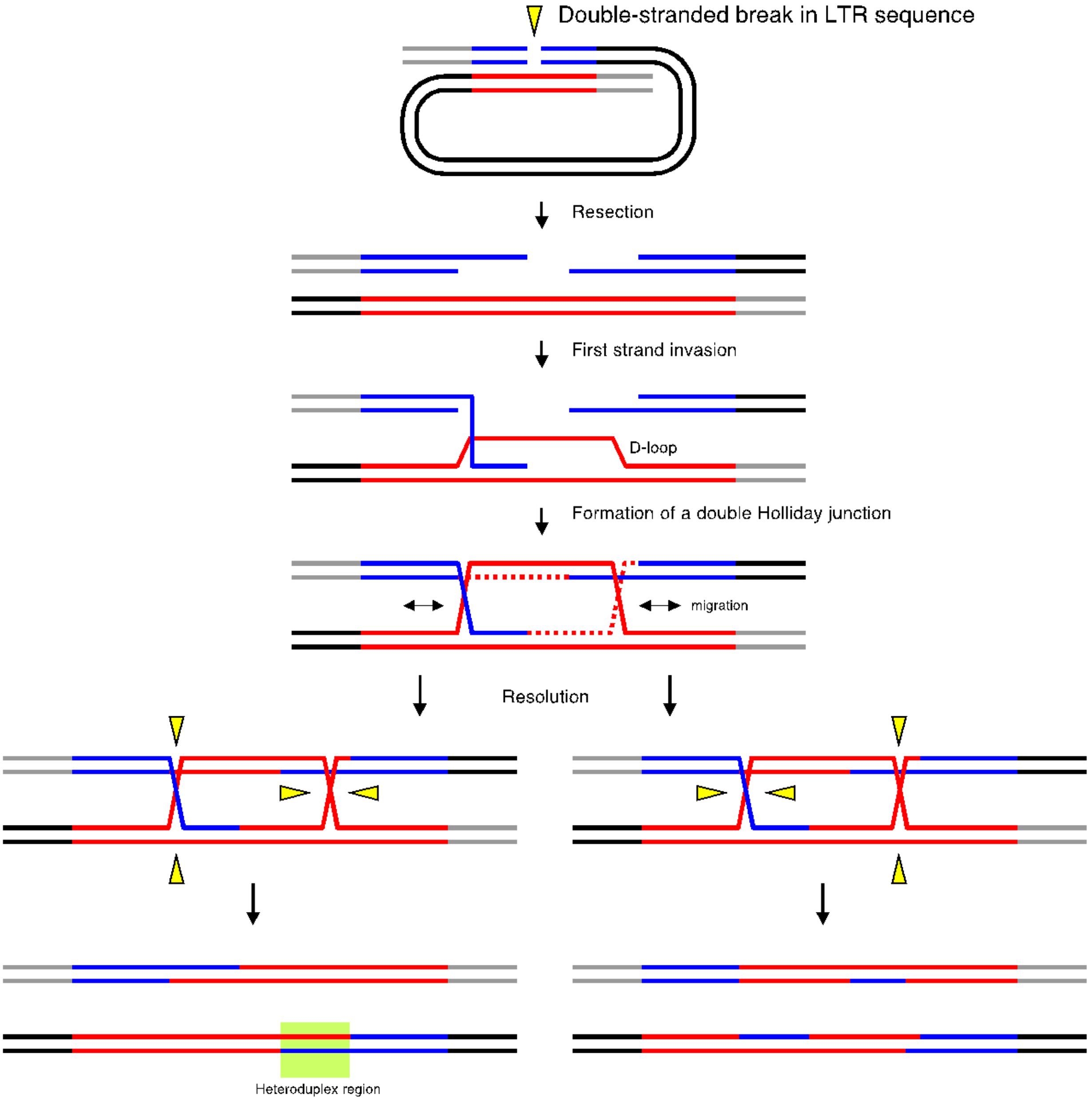

Figure S6 Schematic overview of double Holliday junction formation during illegitimate recombination between two LTR sequences.
Colours are as in Fig. 2. For clarity, the extent of resection is highly exaggerated in the figure.
