# Supplementary material for: Formation of Extrachromosomal Circular DNA from Long Terminal Repeats of Retrotransposons in Saccharomyces cerevisiae
Source: G3 (Bethesda). 2015 Dec 17;6(2):453–62. doi: 10.1534/g3.115.025858 (PMC4751563; doi:10.1534/g3.115.025858)
Supplement: Supporting Information [file supp_g3.115.025858_TableS1.pdf]

Table S1   Coverage of individual full-length Ty element loci

chromosome	start	stop	family	size	number of unique 142-mers in element	Sample S1: percentage of loci covered by reads	Sample S1: percentage of loci covered by uniquely mapping reads	Sample S1: average weighted coverage	Sample S1: average unique coverage	Sample S2: percentage of loci covered by reads	Sample S2: percentage of loci covered by uniquely mapping reads	Sample S2: average weighted coverage
chrI	160239	166163	TY1;Full	5925	404	99.95	6.13	4.19	0.07	99.98	14.46	10.86
chrII	221040	226955	TY1;Full	5916	1391	95.71	26.10	2.34	0.46	98.80	43.26	6.50
chrII	259576	265492	TY1;Full	5917	1002	95.93	0.00	2.34	0.00	97.89	11.93	7.34
chrIV	645508	651417	TY1;Full	5910	199	99.98	0.00	3.55	0.00	99.98	13.89	10.08
chrIV	878301	884218	TY1;Full	5918	693	98.75	0.00	3.41	0.00	99.97	18.96	10.34
chrIV	987155	992639	TY1;Full	5485	197	99.98	2.57	3.56	0.03	99.98	15.33	10.57
chrIV	1095765	1101690	TY1;Full	5926	5	99.95	0.00	4.21	0.00	99.85	0.00	11.00
chrIV	1206697	1212614	TY1;Full	5918	148	99.71	3.89	3.13	0.10	99.81	8.01	9.59
chrV	443393	449316	TY1;Full	5924	4	99.65	0.00	4.08	0.00	99.90	0.00	11.26
chrV	492691	498416	TY1;Full	5726	188	99.88	2.46	2.68	0.02	99.20	4.09	6.97
chrVII	535764	541689	TY1;Full	5926	14	99.95	0.00	3.66	0.00	99.73	7.61	10.19
chrVII	561855	567764	TY1;Full	5910	141	99.98	5.74	3.84	0.20	99.98	13.28	9.70
chrVII	817398	823311	TY1;Full	5914	236	99.88	0.00	3.03	0.00	99.98	11.24	8.87
chrVIII	543610	549637	TY1;Full	6028	521	99.93	3.17	3.04	0.05	99.05	10.02	7.97
chrX	472456	478043	TY1;Full	5588	436	99.96	12.99	4.39	0.91	99.87	23.98	12.79
chrX	478044	483965	TY1;Full	5922	0	99.98	2.25	3.74	0.02	99.92	0.00	10.53
chrXII	215081	221006	TY1;Full	5926	609	98.95	0.00	2.81	0.00	98.63	0.00	7.48
chrXII	475975	481898	TY1;Full	5924	142	99.98	0.00	4.10	0.00	99.97	7.21	11.25
chrXII	593147	599054	TY1;Full	5908	318	99.54	0.00	2.57	0.00	99.98	13.44	8.08
chrXII	650826	656744	TY1;Full	5919	416	99.85	0.00	3.19	0.00	99.86	13.68	8.80
chrXIII	184170	190083	TY1;Full	5914	142	99.98	3.38	4.08	0.06	99.83	5.34	10.90
chrXIII	196332	202234	TY1;Full	5903	155	99.76	0.00	3.96	0.00	99.70	2.76	10.75
chrXIII	357004	362917	TY1;Full	5914	1278	99.24	20.71	2.32	0.60	97.97	37.62	5.11
chrXIII	372696	378621	TY1;Full	5926	75	99.98	2.09	3.29	0.04	99.88	6.99	8.40
chrXIV	96610	102523	TY1;Full	5914	1673	96.96	26.36	2.44	0.52	92.26	21.51	5.45
chrXIV	519164	525053	TY1;Full	5890	1712	96.86	16.01	3.68	1.44	95.99	43.06	10.16
chrXV	117703	123628	TY1;Full	5926	402	99.98	3.09	4.62	0.69	99.80	15.79	12.55
chrXV	594822	600735	TY1;Full	5914	0	99.95	0.00	3.08	0.00	99.81	0.00	8.77
chrXVI	56452	62375	TY1;Full	5924	0	99.98	0.00	4.10	0.00	99.98	6.45	11.44
chrXVI	804651	810558	TY1;Full	5908	0	99.83	0.00	3.63	0.00	99.98	0.00	10.17
chrXVI	844408	850336	TY1;Full	5929	138	99.92	0.00	4.14	0.00	99.85	0.00	10.68
chrXVI	850625	856550	TY1;Full	5926	653	98.77	4.74	4.93	1.79	99.98	30.12	12.64
chrII	29641	35599	TY2;Full	5959	2433	86.39	4.03	0.52	0.05	83.99	0.00	1.04
chrIII	84811	90769	TY2;Full	5959	522	94.95	3.52	0.73	0.05	96.59	9.70	2.16
chrIV	513691	519645	TY2;Full	5955	912	92.07	2.38	0.68	0.02	95.37	3.61	1.65
chrIV	871819	877777	TY2;Full	5959	681	96.19	2.37	0.67	0.02	95.44	4.65	1.70
chrIV	981168	987126	TY2;Full	5959	1344	99.80	31.90	1.83	1.14	99.95	57.93	6.75
chrVI	137908	143866	TY2;Full	5959	1	93.46	0.00	0.68	0.00	98.61	0.00	1.93
chrVII	568745	574704	TY2;Full	5960	698	96.93	4.38	0.72	0.07	96.17	5.12	1.72
chrVII	811452	817397	TY2;Full	5946	12	93.61	0.00	0.69	0.00	98.65	0.00	1.92
chrXII	941190	947148	TY2;Full	5959	689	93.93	0.10	0.65	0.00	93.07	3.78	1.74
chrXII	976255	981697	TY2;Full	5443	690	90.12	8.21	0.66	0.10	93.96	7.35	1.42
chrXIV	562034	567993	TY2;Full	5960	858	97.38	16.75	0.89	0.28	96.41	2.99	1.56
chrXV	704065	710025	TY2;Full	5961	616	92.62	2.26	0.64	0.04	93.63	5.99	1.46
chrXV	970284	976238	TY2;Full	5955	1031	92.56	0.00	0.62	0.00	91.96	0.00	1.54

chrIX	205217	210644	TY3;Full	5428	3201	12.56	2.60	0.06	0.03	8.71	2.49	0.03
chrVII	707199	712549	TY3;Full	5351	3122	20.48	10.54	0.14	0.11	6.04	0.00	0.01
chrVIII	85535	91757	TY4;Full	6223	489	27.30	0.00	0.12	0.00	55.55	3.62	0.39
chrX	197543	203768	TY4;Full	6226	3091	38.13	18.60	0.43	0.31	42.05	7.23	0.26
chrXVI	436887	443109	TY4;Full	6223	314	27.94	0.00	0.11	0.00	60.84	9.05	0.51
chrIII	1179	4322	TY5;Full	3144	3003	12.53	12.53	0.13	0.13	12.72	12.72	0.17

Notes:
Ty elements with at least 99.5% of their entire length covered by sequence reads are highlighted in grey.

Ty elements with no uniquely mapping reads are coloured in blue text if the loci DOES NOT contain any unique 142-mer sequence.

Ty elements with no uniquely mapping reads are coloured in red text if the loci DOES contain unique 142-mer sequence(s).

The full-length Ty1 element at chrX:478044-478043 has no unique 142-mer sequences but nevertheless has uniquely mapping reads. This happens as read are allowed to map partly to Ty loci and partly to genomic surroundings, whereas unique 142-mers are only considering when residing completely within Ty elements.
