# Supplementary material for: Formation of Extrachromosomal Circular DNA from Long Terminal Repeats of Retrotransposons in Saccharomyces cerevisiae
Source: G3 (Bethesda). 2015 Dec 17;6(2):453–62. doi: 10.1534/g3.115.025858 (PMC4751563; doi:10.1534/g3.115.025858)
Supplement: Supporting Information [file supp_g3.115.025858_TableS2.pdf]

Table S2 Overview of samples

Sample	Read length	Number of reads	Strain background	Strain name	Genotype
S1	141.3*	73,049,571	S288c	M3750	MATa ura3 gal
S2	193*	81,221,653			
Z1	141	72,626,304	S288c BY	BY4745	*MATa his3Δ1 leu2Δ0 met15Δ0 ura3Δ0 KanMX::xxxΔ
Z3	141	66,762,805			
R1	225	23,927,042	S288c BY	BY4741	*MATa his3Δ1 leu2Δ0 met15Δ0 ura3Δ0 KanMX::xxxΔ
R3	141	87,929,159			
B02	2x141	10,714,844	S288c BY	UCC5185	MATa/MATα ade2::hisG/ade2::hisG his3/his3 leu2/leu2 LYS2/lys2 ura3Δ0/ura3Δ0 trp1Δ63/trp1Δ63 MET15/met15Δ::ADE2 hoΔ::PSCW11-cre-EBD78-NATMX/hoΔ::PSCW11-creEBD78-NATMX loxP-UBC9-loxP-LEU2/loxP-UBC9-loxP-LEU2 loxP-CDC20-Intron-loxPHPHMX/loxP-CDC20-Intron-loxP-HPHMX
B03	2x141	11,727,134			
B04	2x141	11,973,456			
B05	2x141	11,151,228			
