# Supplementary material for: Formation of Extrachromosomal Circular DNA from Long Terminal Repeats of Retrotransposons in Saccharomyces cerevisiae
Source: G3 (Bethesda). 2015 Dec 17;6(2):453–62. doi: 10.1534/g3.115.025858 (PMC4751563; doi:10.1534/g3.115.025858)
Supplement: Supporting Information [file supp_g3.115.025858_TableS3.pdf]

Table S3   Genomic coordinates of Ty sequences.

chromsomes	start	end	strand	family	type
chrI	22232	22552	+	TY1	S
chrI	138754	138990	-	TY1	S
chrI	160106	160237	-	TY1	S
chrI	160239	166163	-	TY1	F
chrI	182614	182953	+	TY3	S
chrI	183136	183468	+	TY1	S
chrI	189420	189754	+	TY2	S
chrI	209460	209769	-	TY1	S
chrII	8848	9092	+	TY1	S
chrII	9093	9424	+	TY2	S
chrII	9425	9518	+	TY1	S
chrII	29501	29640	+	TY1	S
chrII	29641	35599	+	TY2	F
chrII	35600	35793	+	TY1	S
chrII	35848	36218	-	TY4	S
chrII	197033	197323	-	TY1	S
chrII	197717	198057	+	TY3	S
chrII	198170	198252	-	TY1	S
chrII	220934	221039	+	TY1	S
chrII	221040	226955	+	TY1	F
chrII	258668	258974	-	TY1	S
chrII	259576	265492	+	TY1	F
chrII	266178	266256	-	TY1	S
chrII	327068	327393	+	TY1	S
chrII	327394	327693	+	TY1	S
chrII	350457	350749	+	TY1	S
chrII	460473	460762	+	TY1	S
chrII	643483	643853	-	TY4	S
chrII	643854	643997	+	TY1	S
chrII	644364	644596	-	TY1	S
chrIII	1179	4322	+	TY5	F
chrIII	4480	4580	+	TY5	S
chrIII	82700	83036	+	TY1	S
chrIII	83055	83196	-	TY1	S
chrIII	84069	84294	+	TY1	S
chrIII	84295	84626	+	TY2	S
chrIII	84627	84718	+	TY1	S
chrIII	84811	90769	+	TY2	F
chrIII	124132	124463	-	TY1	S
chrIII	142790	143078	-	TY1	S
chrIII	149480	149787	+	TY1	S
chrIII	150218	150540	+	TY1	S
chrIII	150693	151051	-	TY4	S
chrIII	151465	151518	-	TY1	S
chrIII	151519	151849	+	TY1	S
chrIII	168387	168605	+	TY3	S
chrIII	168606	168929	-	TY1	S
chrIII	168930	169064	+	TY3	S
chrIII	169203	169540	+	TY1	S

chrIII	169569	169884	+	TY1	S
chrIII	227660	227810	-	TY1	S
chrIII	291373	291709	+	TY1	S
chrIII	291919	292164	+	TY5	S
chrIII	292288	292525	+	TY5	S
chrIII	295000	295317	+	TY1	S
chrIII	295856	295926	+	TY1	S
chrIII	295955	296187	-	TY1	S
chrIV	3336	3461	+	TY5	S
chrIV	8080	8420	+	TY4	S
chrIV	83292	83483	+	TY3_1p	S
chrIV	359317	359480	-	TY1	S
chrIV	434421	434737	-	TY1	S
chrIV	434740	434944	-	TY1	S
chrIV	434949	435096	+	TY1	S
chrIV	437147	437516	+	TY4	S
chrIV	437520	437697	-	TY1	S
chrIV	512986	513256	-	TY1	S
chrIV	513257	513588	-	TY1	S
chrIV	513589	513641	-	TY1	S
chrIV	513691	519645	-	TY2	F
chrIV	519942	520157	+	TY1	S
chrIV	520180	520474	-	TY1	S
chrIV	520475	520794	+	TY2	S
chrIV	620559	620662	-	TY1	S
chrIV	645238	645507	-	TY3	S
chrIV	645508	651417	-	TY1	F
chrIV	651418	651501	-	TY3	S
chrIV	651688	651956	-	TY1	S
chrIV	668094	668434	+	TY3	S
chrIV	668624	668782	+	TY1	S
chrIV	802903	805652	+	TY1	T
chrIV	805756	805972	-	TY1	S
chrIV	871420	871742	+	TY1	S
chrIV	871747	871818	-	TY1	S
chrIV	871819	877777	+	TY2	F
chrIV	877778	878042	-	TY1	S
chrIV	878062	878300	+	TY1	S
chrIV	878301	884218	-	TY1	F
chrIV	945348	945670	+	TY1	S
chrIV	945698	945757	-	TY1	S
chrIV	945952	946292	-	TY3	S
chrIV	981168	987126	+	TY2	F
chrIV	987155	992639	-	TY1	F
chrIV	992640	992739	+	TY1	S
chrIV	992740	992812	-	TY3	S
chrIV	1017553	1017675	-	TY1	S
chrIV	1017678	1018020	-	TY4	S
chrIV	1018212	1018366	+	TY4	S
chrIV	1018389	1018514	+	TY1	S
chrIV	1023134	1023474	+	TY3	S
chrIV	1095639	1095764	-	TY1	S
chrIV	1095765	1101690	+	TY1	F

chrIV	1101691	1101876	-	TY1	S
chrIV	1151034	1151363	+	TY1	S
chrIV	1151391	1151511	-	TY1	S
chrIV	1174832	1174952	+	TY1	S
chrIV	1175129	1175310	-	TY3	S
chrIV	1175311	1175642	+	TY1	S
chrIV	1175643	1175807	-	TY3	S
chrIV	1201081	1201275	+	TY1	S
chrIV	1201450	1201590	+	TY1	S
chrIV	1206697	1212614	+	TY1	F
chrIV	1305352	1305442	-	TY3	S
chrIV	1352637	1352968	+	TY2	S
chrIV	1352969	1353219	+	TY1	S
chrIV	1461957	1462025	+	TY1	S
chrIV	1516337	1516421	-	TY2	T
chrIV	1518469	1518787	+	TY1	S
chrIX	196650	197020	-	TY4	S
chrIX	197137	197485	-	TY1	S
chrIX	205217	210644	+	TY3	F
chrIX	246217	246549	+	TY2	S
chrIX	300407	300725	-	TY1	S
chrIX	301656	301730	+	TY1	S
chrIX	324389	324732	+	TY3	S
chrIX	324829	325162	+	TY1	S
chrIX	325163	325216	+	TY1	S
chrIX	336576	336892	-	TY1	S
chrIX	426198	426422	-	TY1	S
chrV	6986	7117	+	TY5	S
chrV	9631	9738	-	TY2	T
chrV	62142	62474	+	TY2	S
chrV	62475	62756	+	TY1	S
chrV	99303	99660	-	TY1	S
chrV	118227	118556	-	TY4	S
chrV	135608	135939	-	TY2	S
chrV	136063	136183	+	TY1	S
chrV	137324	137612	+	TY1	S
chrV	138221	138553	+	TY1	S
chrV	176349	176574	+	TY1	S
chrV	176575	176714	-	TY1	S
chrV	176715	176845	+	TY1	S
chrV	248828	249107	+	TY1	S
chrV	249108	249406	-	TY1	S
chrV	249444	249751	+	TY1	S
chrV	249929	250269	-	TY3	S
chrV	287771	288056	-	TY1	S
chrV	288643	288724	+	TY1	S
chrV	312206	312261	+	TY3_1p	S
chrV	312272	312437	+	TY1	S
chrV	314079	314424	+	TY4	S
chrV	354134	354355	+	TY1	S
chrV	354356	354726	-	TY4	S
chrV	354727	354836	+	TY1	S
chrV	354847	354913	-	TY3	S

chrV	431486	431818	-	TY2	S
chrV	434627	434967	-	TY3	S
chrV	435066	435122	+	TY1	S
chrV	435124	435438	-	TY1	S
chrV	435942	436273	-	TY2	S
chrV	436275	436601	-	TY4	S
chrV	436602	436633	+	TY1	S
chrV	438522	438584	-	TY1	S
chrV	442730	443068	+	TY1	S
chrV	443366	443392	+	TY2	S
chrV	443393	449316	-	TY1	F
chrV	449317	449626	+	TY2	S
chrV	449736	450035	+	TY1	S
chrV	469326	469388	-	TY1	S
chrV	487829	488160	+	TY2	S
chrV	492434	492690	-	TY3	S
chrV	492691	498416	-	TY1	F
chrV	498417	498505	-	TY3	S
chrV	551437	551759	+	TY1	S
chrV	552003	552216	-	TY1	S
chrV	566695	566945	-	TY5	S
chrV	568786	569017	+	TY5	S
chrVI	100575	100902	+	TY1	S
chrVI	119074	119171	+	TY1	S
chrVI	137658	137907	+	TY1	S
chrVI	137908	143866	+	TY2	F
chrVI	143867	143937	+	TY1	S
chrVI	144200	144518	+	TY4	S
chrVI	144534	144815	-	TY1	S
chrVI	158354	158666	+	TY1	S
chrVI	161532	161691	+	TY1	S
chrVI	161808	162129	-	TY1	S
chrVI	181388	181471	+	TY1	S
chrVI	181739	181884	+	TY1	S
chrVI	191671	191834	-	TY1	S
chrVI	205102	205241	+	TY3_1p	S
chrVI	205256	205503	-	TY1	S
chrVI	206910	207126	+	TY1	S
chrVI	261283	261475	+	TY1	S
chrVII	863	1079	+	TY5	S
chrVII	1080	1239	+	TY5	S
chrVII	110811	110858	-	TY2	S
chrVII	110859	111229	+	TY4	S
chrVII	111230	111509	-	TY2	S
chrVII	111510	111702	+	TY1	S
chrVII	114943	115273	+	TY1	S
chrVII	121168	121340	+	TY1	S
chrVII	122093	122145	-	TY1	S
chrVII	204990	205343	+	TY4	S
chrVII	287945	288115	+	TY1	S
chrVII	318686	318826	+	TY1	S
chrVII	318827	318984	-	TY4	S
chrVII	319033	319344	-	TY1	S

chrVII	319426	319765	-	TY3	S
chrVII	320358	320710	-	TY4	S
chrVII	327132	327346	-	TY1	S
chrVII	327907	328220	+	TY1	S
chrVII	328230	328503	+	TY1	S
chrVII	401619	401897	+	TY3	S
chrVII	401962	402293	+	TY1	S
chrVII	405007	405148	+	TY2	S
chrVII	405149	405210	+	TY2	S
chrVII	405231	405456	-	TY3	S
chrVII	412458	412727	+	TY1	S
chrVII	412819	412887	+	TY3_1p	S
chrVII	535215	535546	+	TY1	S
chrVII	535548	535763	-	TY1	S
chrVII	535764	541689	+	TY1	F
chrVII	541690	541786	-	TY1	S
chrVII	561855	567764	-	TY1	F
chrVII	568168	568287	+	TY3_1p	S
chrVII	568745	574704	-	TY2	F
chrVII	660647	660936	-	TY1	S
chrVII	701114	701354	+	TY1	S
chrVII	701356	701546	+	TY1	S
chrVII	707199	712549	+	TY3	F
chrVII	712900	713081	+	TY4	S
chrVII	713088	713388	-	TY1	S
chrVII	735492	735546	-	TY1	S
chrVII	735549	735877	+	TY1	S
chrVII	735987	736327	+	TY3	S
chrVII	738565	738604	+	TY1	S
chrVII	738741	739040	+	TY1	S
chrVII	774041	774167	+	TY1	S
chrVII	778789	779117	+	TY2	S
chrVII	779118	779236	+	TY1	S
chrVII	779237	779520	-	TY1	S
chrVII	793244	793564	+	TY1	S
chrVII	811101	811238	-	TY4	S
chrVII	811452	817397	+	TY2	F
chrVII	817398	823311	-	TY1	F
chrVII	845286	845635	+	TY3	S
chrVII	875350	875666	+	TY1	S
chrVII	875956	876284	+	TY1	S
chrVII	879030	879240	+	TY4	S
chrVII	879273	879464	+	TY1	S
chrVII	931174	931543	-	TY4	S
chrVII	931691	932022	+	TY1	S
chrVIII	7993	8224	-	TY5	S
chrVIII	13261	13324	-	TY5	S
chrVIII	85384	85534	-	TY3	S
chrVIII	85535	91757	+	TY4	F
chrVIII	91773	92095	-	TY2	S
chrVIII	92272	92386	+	TY1	S
chrVIII	116416	116747	-	TY1	S
chrVIII	116767	117054	-	TY1	S

chrVIII	133198	133395	-	TY1	S
chrVIII	133568	133679	-	TY1	S
chrVIII	133680	134002	+	TY1	S
chrVIII	134003	134217	-	TY1	S
chrVIII	146324	146664	-	TY3	S
chrVIII	146837	147705	+	TY4	T
chrVIII	238007	238205	+	TY1	S
chrVIII	238305	238394	+	TY4	S
chrVIII	358685	358852	-	TY1	S
chrVIII	358853	359070	-	TY4	S
chrVIII	359071	359477	-	TY1	S
chrVIII	359082	359346	+	TY1	S
chrVIII	389180	389512	-	TY2	S
chrVIII	389513	389584	+	TY1	S
chrVIII	389630	389847	-	TY1	S
chrVIII	463920	464147	-	TY1	S
chrVIII	466423	466474	+	TY3_1p	S
chrVIII	466556	466863	+	TY1	S
chrVIII	466881	466975	-	TY3	S
chrVIII	530070	530379	-	TY1	S
chrVIII	543610	549637	-	TY1	F
chrX	29187	29409	+	TY1	S
chrX	59504	59783	+	TY1	S
chrX	197399	197492	+	TY3	S
chrX	197493	197542	+	TY1	S
chrX	197543	203768	+	TY4	F
chrX	203769	204392	+	TY1	S
chrX	203793	204114	-	TY2	S
chrX	354539	354838	-	TY1	S
chrX	354839	355170	-	TY2	S
chrX	374768	374835	-	TY4	S
chrX	374849	374902	-	TY1	S
chrX	377539	377590	+	TY1	S
chrX	377600	377854	-	TY1	S
chrX	378009	378279	-	TY3_1p	S
chrX	416260	416425	+	TY4	S
chrX	416504	417018	+	TY1	S
chrX	421918	422290	+	TY4	S
chrX	422588	422881	+	TY1	S
chrX	472456	478043	+	TY1	F
chrX	478044	483965	+	TY1	F
chrX	517951	518091	+	TY1	S
chrX	531508	531757	-	TY1	S
chrX	537733	538027	-	TY1	S
chrX	538096	538427	+	TY2	S
chrX	540759	541079	+	TY1	S
chrX	541088	541420	-	TY2	S
chrX	543441	543762	+	TY1	S
chrX	737371	737678	-	TY4	S
chrX	742331	742456	-	TY5	S
chrXI	74241	74523	+	TY1	S
chrXI	83813	84120	-	TY1	S
chrXI	141176	141294	-	TY1	S

chrXI	162836	163174	+	TY1	S
chrXI	163175	163394	-	TY4	S
chrXI	202288	202627	-	TY3	S
chrXI	219058	219367	-	TY1	S
chrXI	301950	301983	+	TY1	S
chrXI	301984	302269	-	TY1	S
chrXI	313247	313562	-	TY1	S
chrXI	313837	314158	+	TY2	S
chrXI	378914	378980	-	TY3	S
chrXI	378991	379112	+	TY1	S
chrXI	379113	379265	-	TY3_1p	S
chrXI	457783	458114	-	TY1	S
chrXI	489929	490057	-	TY1	S
chrXI	490107	490430	+	TY1	S
chrXI	490431	490489	+	TY1	S
chrXI	513174	513483	-	TY1	S
chrXI	517095	517426	-	TY1	S
chrXI	664814	664914	-	TY5	S
chrXI	665068	665306	+	TY5	S
chrXII	91573	91822	-	TY4	S
chrXII	91959	92048	-	TY1	S
chrXII	92049	92380	-	TY1	S
chrXII	92381	92480	-	TY1	S
chrXII	168042	168379	-	TY3	S
chrXII	168542	168649	+	TY1	S
chrXII	168650	168732	-	TY1	S
chrXII	215081	221006	-	TY1	F
chrXII	221008	221307	+	TY1	S
chrXII	365832	366145	-	TY1	S
chrXII	373480	373534	-	TY1	S
chrXII	373770	373969	-	TY1	S
chrXII	374000	374339	+	TY3	S
chrXII	426829	427002	-	TY3_1p	S
chrXII	448843	449114	+	TY1	S
chrXII	475975	481898	-	TY1	F
chrXII	592708	593037	-	TY1	S
chrXII	593147	599054	+	TY1	F
chrXII	599171	599269	-	TY4	S
chrXII	599270	599518	+	TY1	S
chrXII	599519	599831	-	TY4	S
chrXII	599843	599913	+	TY1	S
chrXII	650826	656744	+	TY1	F
chrXII	657024	657366	+	TY3	S
chrXII	657451	657769	+	TY1	S
chrXII	688131	688363	-	TY2	S
chrXII	688371	688666	+	TY1	S
chrXII	731680	732002	-	TY2	S
chrXII	734384	734673	-	TY1	S
chrXII	784208	784288	-	TY1	S
chrXII	793552	793870	+	TY3	S
chrXII	796764	797088	+	TY1	S
chrXII	817840	818072	+	TY4	S
chrXII	818073	818403	-	TY1	S

chrXII	941190	947148	+	TY2	F
chrXII	963217	963535	+	TY1	S
chrXII	976255	981697	-	TY2	F
chrXII	1052301	1052630	-	TY1	S
chrXII	1052660	1052754	-	TY1	S
chrXIII	131989	132317	-	TY1	S
chrXIII	167945	168262	-	TY1	S
chrXIII	168349	168681	-	TY2	S
chrXIII	184170	190083	+	TY1	F
chrXIII	196332	202234	+	TY1	F
chrXIII	259596	259901	-	TY1	S
chrXIII	289947	290015	-	TY1	S
chrXIII	290048	290223	-	TY4	S
chrXIII	290441	290521	+	TY1	S
chrXIII	290583	290660	-	TY1	S
chrXIII	321342	321527	-	TY4	S
chrXIII	357004	362917	-	TY1	F
chrXIII	372626	372695	+	TY1	S
chrXIII	372696	378621	-	TY1	F
chrXIII	378622	379197	+	TY1	S
chrXIII	378731	379060	-	TY1	S
chrXIII	420973	421316	-	TY1	S
chrXIII	462885	463103	-	TY1	S
chrXIII	463140	463460	-	TY1	S
chrXIII	481213	481544	-	TY1	S
chrXIII	481570	481807	+	TY1	S
chrXIII	503191	503381	+	TY1	S
chrXIII	503664	503766	+	TY1	S
chrXIII	503770	504140	+	TY4	S
chrXIII	504242	504344	+	TY4	S
chrXIII	504345	504676	+	TY1	S
chrXIII	504677	504781	+	TY4	S
chrXIII	573111	573337	-	TY1	S
chrXIII	573356	573445	-	TY1	S
chrXIII	748025	748124	+	TY1	S
chrXIII	768547	768878	+	TY2	S
chrXIII	768879	769211	+	TY1	S
chrXIII	808536	808905	-	TY4	S
chrXIII	808999	809232	+	TY1	S
chrXIII	837476	837753	+	TY1	S
chrXIII	837754	837904	+	TY3	S
chrXIV	96451	96609	+	TY4	S
chrXIV	96610	102523	-	TY1	F
chrXIV	102549	102631	+	TY1	S
chrXIV	102632	102700	-	TY3	S
chrXIV	102701	104702	+	TY1	S
chrXIV	519164	525053	+	TY1	F
chrXIV	546424	546685	+	TY3_1p	S
chrXIV	546739	547078	+	TY3	S
chrXIV	552282	552482	-	TY1	S
chrXIV	552800	553128	+	TY1	S
chrXIV	561378	561486	+	TY1	S
chrXIV	561500	561606	-	TY1	S

chrXIV	561608	561978	+	TY4	S
chrXIV	562034	567993	-	TY2	F
chrXIV	631937	631999	+	TY3_1p	S
chrXIV	632007	632112	-	TY1	S
chrXIV	632121	632491	-	TY4	S
chrXIV	726235	726575	+	TY3	S
chrXIV	726615	726946	-	TY2	S
chrXIV	726952	727214	+	TY3_1p	S
chrXIV	727364	727667	-	TY1	S
chrXV	2894	3223	-	TY1	S
chrXV	3809	3986	+	TY4	S
chrXV	16739	16933	+	TY1	S
chrXV	18163	18259	-	TY1	S
chrXV	18301	18554	-	TY1	S
chrXV	33629	33930	+	TY1	S
chrXV	113294	113626	-	TY1	S
chrXV	113685	113752	-	TY1	S
chrXV	117703	123628	+	TY1	F
chrXV	226696	227037	+	TY3	S
chrXV	227096	227234	+	TY1	S
chrXV	227693	227860	+	TY1	S
chrXV	227861	228178	-	TY1	S
chrXV	228179	228263	+	TY1	S
chrXV	300235	300441	-	TY1	S
chrXV	300686	301023	-	TY1	S
chrXV	340600	340900	-	TY1	S
chrXV	354129	354467	+	TY3	S
chrXV	354682	354959	+	TY1	S
chrXV	355118	355188	+	TY1	S
chrXV	436924	437008	+	TY1	S
chrXV	437304	438486	-	TY1	T
chrXV	463542	463647	+	TY1	S
chrXV	571361	571462	-	TY4	S
chrXV	571491	571808	+	TY1	S
chrXV	594514	594813	+	TY1	S
chrXV	594822	600735	+	TY1	F
chrXV	664004	664326	-	TY1	S
chrXV	664330	664465	+	TY1	S
chrXV	664815	665147	+	TY2	S
chrXV	697504	697770	-	TY1	S
chrXV	697810	697906	-	TY4	S
chrXV	703158	703382	-	TY3	S
chrXV	703431	703662	-	TY1	S
chrXV	703671	703754	+	TY1	S
chrXV	703774	704064	+	TY4	S
chrXV	704065	710025	-	TY2	F
chrXV	710026	710109	+	TY4	S
chrXV	854275	854614	+	TY3	S
chrXV	968989	969299	+	TY3	S
chrXV	969300	969429	+	TY1	S
chrXV	969430	976400	+	TY3	S
chrXV	970284	976238	+	TY2	F
chrXV	976257	976336	-	TY1	S

chrXV	979867	980140	+	TY1	S
chrXV	980257	980588	-	TY1	S
chrXVI	55663	55858	-	TY1	S
chrXVI	56290	56356	+	TY3	S
chrXVI	56357	56451	+	TY1	S
chrXVI	56452	62375	+	TY1	F
chrXVI	62376	63006	+	TY1	S
chrXVI	62389	62720	+	TY2	S
chrXVI	210450	210770	+	TY1	S
chrXVI	211576	211907	+	TY2	S
chrXVI	436058	436246	+	TY1	S
chrXVI	436247	436584	-	TY1	S
chrXVI	436585	436728	+	TY1	S
chrXVI	436887	443109	-	TY4	F
chrXVI	560478	560809	+	TY1	S
chrXVI	560810	561081	+	TY1	S
chrXVI	561165	561320	-	TY3_1p	S
chrXVI	571693	571834	+	TY1	S
chrXVI	571842	572170	-	TY1	S
chrXVI	689059	689439	-	TY1	S
chrXVI	769317	769657	+	TY3	S
chrXVI	769801	769897	+	TY1	S
chrXVI	775858	776090	+	TY3_1p	S
chrXVI	776091	776401	+	TY1	S
chrXVI	776402	776502	+	TY3_1p	S
chrXVI	776665	776854	-	TY1	S
chrXVI	777842	778152	+	TY1	S
chrXVI	780918	781217	-	TY1	S
chrXVI	804495	804634	+	TY1	S
chrXVI	804651	810558	-	TY1	F
chrXVI	844355	844407	-	TY1	S
chrXVI	844408	850336	+	TY1	F
chrXVI	850337	850508	-	TY1	S
chrXVI	850625	856550	-	TY1	F
chrXVI	856551	856881	+	TY3	S
chrXVI	880382	880722	+	TY3	S
chrXVI	880770	880965	-	TY3_1p	S
chrXVI	881483	881826	-	TY4	S
chrXVI	933067	933400	+	TY4	S
chrXVI	933539	933675	+	TY1	S
chrXVI	937876	937992	-	TY1	S

Taken from Carr et al. with few adjustments.
Types denotes full-length (F), solo LTR (S), or truncated element (T)
